# Colchicin – Phönix aus der Asche

**DOI:** 10.1007/s00508-024-02490-7

**Published:** 2025-02-06

**Authors:** Raimund Lunzer, Georg Delle-Karth, Markus Zeitlinger, Marlene Prager, Lena Maria Pracher

**Affiliations:** 1 Abteilung für Innere Medizin II, Krankenhaus der Barmherzigen Brüder, Marschallgasse 12, 8020 Graz, Österreich; 2 Abteilung für Kardiologie, Klinik Floridsdorf, Wien, Österreich; 3 Universitätsklinik für Klinische Pharmakologie, Medizinische Universität Wien, Wien, Österreich

**Keywords:** Gicht, Kardiovaskulär, Arteriosklerose, Myocard, Familiäres Mittelmeerfieber, Gout, Cardiovascular, Arteriosclerosis, Myocardial infarction, Familial Mediterranean fever

## Abstract

Colchicin ist ein entzündungshemmender pflanzlicher Arzneistoff mit einer jahrtausendealten Geschichte. Es wird seit jeher erfolgreich in der Akuttherapie und Prophylaxe der Gicht eingesetzt und konnte sich einen festen Platz im pharmakologischen Standardrepertoire bei familiärem Mittelmeerfieber, Perikarditis, neutrophilen Dermatosen, Morbus Behçet und oralen therapierefraktären schweren Aphthosen sichern. Rezent hat die US-amerikanische Food and Drug Administration (FDA) Colchicin zugelassen, um das Risiko von Myokardinfarkt, Schlaganfall, koronarer Revaskularisation und kardiovaskulärem Tod bei erwachsenen Patienten mit bestehender atherosklerotischer Erkrankung oder mit mehreren Risikofaktoren für eine kardiovaskuläre Erkrankung zu verringern. Der Empfehlungsgrad zur kardiovaskulären Prophylaxe wurde in den aktuellen ESC-Leitlinien von 2024 von IIb auf IIa angehoben. Klinische Studien der vergangenen Jahre belegen ferner einen Effekt beim akuten Koronarsyndrom und Vorhofflimmern. Diese Übersichtsarbeit beleuchtet das Wirksamkeits- und Sicherheitsprofil von Colchicin und bietet einen Einblick in rezente und mögliche zukünftige evidenzbasierte Anwendungsgebiete.

## Abstract (en)

Colchicine is an anti-inflammatory herbal medicine with a history stretching back thousands of years. It is a cornerstone in the acute and prophylactic treatment of gout and has secured a permanent place in the standard pharmacological repertoire for familial Mediterranean fever, pericarditis, neutrophilic dermatoses, Behçet’s disease and severe aphthous ulcers refractory to oral treatment. The US Food and Drug Administration (FDA) has recently approved colchicine to reduce the risk of myocardial infarction, stroke, coronary revascularization and cardiovascular death in adult patients with established atherosclerotic disease or with multiple risk factors for cardiovascular diseases. The recommendation level for cardiovascular prophylaxis was raised from IIb to IIa in the current European Society of Cardiology (ESC) guidelines from 2024. Clinical studies in recent years also demonstrated an effect for acute coronary syndrome and atrial fibrillation. This review article highlights the efficacy and safety profile of colchicine and provides insights into recent and potential future evidence-based fields of application.

Colchicin ist ein trizyklisches, fettlösliches Alkaloid und natürlicher Inhaltsstoff der Herbstzeitlosen (*Colchicum autumnale*) [1]. Die Substanz ist in allen Pflanzenteilen – besonders den Blüten, Samen und Knollen – enthalten und eines der ältesten Heilmittel, die immer noch verwendet und umfassend erforscht werden [2]. Im Lauf der Geschichte wurde Colchicin mit vielen Namen bedacht, darunter Colchicum, Colchicon, Hermodactyl, Surugen und Ephemeron [3]. Die älteste Aufzeichnung stammt aus dem Papyrus von Ebers (ca. 1500 v. Chr.) [4]. Dieser ägyptische Text beschreibt die Herbstzeitlose zur Behandlung von Schmerzen, Schwellungen und Rheuma [6]. Auch den alten Griechen war Colchicin ein Begriff. Theophrastus von Eresos (ca. 371–287 v. Chr.), ein Schüler des Aristoteles, bezeichnet es in seinem Werk *Historia Plantarum* als tödliches Gift mit verzögertem Wirkeintritt und hebt damit dessen kathartische Wirkung hervor [6] – einen Aspekt, den auch der griechische Dichter und Arzt Nicander (ca. 150 v. Chr.) in seinem Buch über Pflanzengifte aufgreift [6]. Ihm zufolge leitet sich das Wort Colchicin vom legendären antiken Königreich Kolchis am Schwarzen Meer ab, wo die Herbstzeitlose in Hülle und Fülle wuchs [6]. Die gleichnamige Hauptstadt spielt in der griechischen Mythologie eine herausragende Rolle [6]. Hier sichert sich Jason das Goldene Vlies, lernt Medea, die Zaubertochter des Königs von Kolchis, kennen und heiratet sie [6]. Mit ihren lebensspendenden Tränken verjüngt sie seinen alternden Vater, vergiftet jedoch anschließend ihre Söhne, nachdem Jason sie für eine andere Frau verraten hatte [6].

Dieser etwas bittere Beigeschmack ändert sich im ersten Jahrhundert nach Christus, als Pedanius Dioscorides Colchicum-Extrakt in *De Mataria Medica* als wirksames Heilmittel gegen Gicht auflistet [6]. Die Anwendung in Form der unterirdischen Colchicum-Knollen überdauert die Zeit und erfreut sich im Mittelalter, speziell im arabischen Raum, großer Beliebtheit [6]. In der westlichen Welt ist der medizinische Gebrauch v. a. Baron Anton von Störck (1731–1803), Leibarzt der Kaiserin von Österreich, zu verdanken [6]. Von Störck führt Experimente an Hunden durch, um Dosierungsprotokolle für Colchicin zu erstellen [6]. Einer seiner Patienten, Nicolas Husson, entwickelt 1783 „Eau Medicinale“, dessen Hauptbestandteil Colchicin ist [6]. Benjamin Franklin, ein langjähriger Gichtkranker, ist von der Wirkung dermaßen begeistert, dass er es bei seiner Rückreise nach Amerika mit im Gepäck hat [6].

Heute wird Colchicin gegen eine Vielzahl weiterer Krankheitsbilder eingesetzt. So ist es fester Bestandteil der Therapie des familiären Mittelmeerfiebers (FMF) und konnte sich als Erstlinientherapie bei akuter und rezidivierender Perikarditis etablieren [7, 8]. Darüber hinaus werden mit Colchicin im Off-Label-Use unter anderem erfolgreich chronisch kutane Vaskulitis, Sweet-Syndrom und Morbus Behçet behandelt [7]. Neuere Daten zeigen einen Nutzen in der Prävention von Vorhofflimmern nach Herzoperationen und Katheterelektrodenablation, bei In-Stent-Restenosen, in der Sekundärprävention von Patienten mit stabiler koronarer Herzkrankheit (KHK), nach einem akuten Koronarsyndrom (ACS), zur Schlaganfallprophylaxe, Herzinsuffizienz und Diabetes mellitus [9, 10]. Diese Übersichtsarbeit soll rezente und mögliche zukünftige evidenzbasierte Anwendungsgebiete abbilden und das Wirksamkeits- und Sicherheitsprofil von Colchicin zusammenfassen.

## Allgemeine Pharmakologie

Oral verabreichtes Colchicin wird im Jejunum und Ileum resorbiert [11]. Die höchste Serumkonzentration erreichen Gesunde in den ersten 2 h [11]. Anschließend fällt der Plasmaspiegel ab, um später aufgrund der enterohepatischen Rezirkulation erneut anzusteigen [11]. Interessanterweise reichert sich Colchicin stark in neutrophilen Granulozyten an, vermutlich da diese Immunzellen nur geringe Mengen von P‑Glykoprotein (P-gp) exprimieren [12]. P‑gp bzw. „multiple drug resistant 1“ (MDR1) gehört zur Familie der ABC-Transporter [13]. Diese Membranproteine besitzen eine ATP-bindende Kassette als gemeinsames Strukturelement und transportieren Xenobiotika aktiv von intra- nach extrazellulär [13]. Die Ausscheidung von Colchicin erfolgt hauptsächlich über die Galle und den Stuhl [11]. Rund 10–20 % der eingenommenen Dosis werden via Zytochrom P450 3A4 (CYP3A4) zu den inaktiven Metaboliten 2‑ und 3‑O-Demethylcolchicin biotransformiert und renal eliminiert [14].

Hepatobiliäre Dysfunktionen, stark eingeschränkte Nierenfunktion sowie Arzneimittelwechselwirkungen mit P‑gp und CYP3A4 sind in der Lage, den Plasmaspiegel von Colchicin zu beeinflussen [14]. In einer Studie, die Serum-Colchicin-Konzentrationen zu verschiedenen Zeitpunkten nach oraler Gabe mithilfe eines spezifischen Radioimmunoassays bestimmte, war die Halbwertszeit bei schwerer Niereninsuffizienz 3‑mal länger als bei Nierengesunden [15]. Bei Patienten mit kombinierter Nieren- und Leberinsuffizienz dauerte es sogar 10-mal so lange, bis Colchicin den Körper wieder verlassen hatte [15]. Dies erklärt, wieso Patienten mit leichten bis mittelschweren Leber- oder Nierenfunktionsstörungen, die gleichzeitig mit einem P‑gp- oder starkem CYP3A4-Inhibitor behandelt werden, unter Colchicin-Therapie sorgfältig überwacht werden sollen [16]. Vorsicht ist zudem geboten bei Mutationen, die das Potenzial haben, die Expression und Transportfunktion von P‑gp zu verändern [17]. Derartige genetische Alterationen werden für das mangelnde Ansprechen auf Colchicin bei FMF verantwortlich gemacht [17]. Ihre praktische Relevanz könnte künftig mit der steigenden Verfügbarkeit pharmakogenetischer Testungen zunehmen. Intravenöse Colchicin-Formulierungen sind nicht mehr gebräuchlich und wurden wegen vermehrt auftretender Nebenwirkungen vom Markt genommen [18].

## Interaktionspotenzial

In Anbetracht der beschriebenen Pharmakologie können starke CYP3A4- und P‑gp-Inhibitoren die Toxizität von Colchicin erhöhen [1, 16]. Wechselwirkungen dieser Art sind abhängig von der Colchicin-Dosis, der Leber- und Nierenfunktion und zusätzlich verordneten Medikamenten. Starke CYP3A4- und P‑gp-Inhibitoren fördern schon bei sehr niedrigen Colchicin-Dosen klinisch relevante Arzneimittelinteraktionen [1]. Im Allgemeinen ist bei Verwendung von CYP3A4-Inhibitoren von einer Verdopplung und bei P‑gp-Inhibitoren von einer Vervierfachung der Colchicin-Plasmakonzentration auszugehen [19]. Geläufige P‑gp-Inhibitoren sind Amiodaron, Carvedilol, Clarithromycin, Itraconazol, Quinidin, Ranolazin, Ritonavir und Verapamil [16, 20]. Zu den starken CYP3A4-Inhibitoren gehören Clarithromycin, Cobicistat, Diltiazem, Itraconazol, Ketoconazol, Ritonavir, Telithromycin und Voriconazol [16, 20]. Moderate CYP3A4-Hemmstoffe inkludieren Ciprofloxacin, Ciclosporin, Erythromycin, Fluconazol, Fluvoxamin und Verapamil [16, 20]. Die simultane Gabe kann die Colchicin-Toxizität verstärken und zu Nebenwirkungen wie Fieber, Erbrechen, Durchfall, Bauchschmerzen, Myalgien und Blutbildschäden führen [16, 20].

Gegenmaßnahmen sind eine Dosisreduktion von Colchicin um bis zu 66 % im Akutfall und bis zu 75 % für die Prophylaxe, eine Verlängerung des Dosierungsintervalls und ggf. eine Verschiebung des Einnahmezeitpunktes [21]. Verapamil und Diltiazem erfordern keine Dosisanpassung im Akutfall, jedoch eine 50- bis 75 %ige Dosisreduktion in der Prophylaxe [21]. Für Ciclosporin wird eine Dosisreduktion von Colchicin um 50 % (akut) bzw. 75 % (Prophylaxe) oder eine Verlängerung des Dosierungsintervalls empfohlen (Prophylaxe alle 2 Tage) [21]. Bei Makrolidantibiotika ist Azithromycin der Vorzug zu geben [21]. Sollte das nicht möglich sein, ist es ratsam, Colchicin 2 Tage vor der ersten Antibiotikagabe abzusetzen bzw. in ausgewählten Fällen die Dosis zu verringern oder das Intervall zu verlängern [21]. Laut Fachinformation können Statine (außer Pravastatin und Rosuvastatin) das Risiko für Myopathien und Rhabdomyolysen mit Nierenversagen erhöhen [16]. Im Rahmen der Sekundärprävention kardiovaskulärer Ereignisse erwies sich Colchicin (0,5 mg/Tag) als Add-on zu Statinen bei Patienten mit stabiler KHK als sicher – es wurden keine Interaktionen beschrieben [22]. Patienten mit Leber- und Nierenfunktionsstörungen sind für Arzneimittelwechselwirkungen besonders prädestiniert. Eine gemeinsame Anwendung von Colchicin und P‑gp-Hemmern und/oder starken CYP3A4-Hemmern ist zu vermeiden, da es schwierig ist, die systemische Exposition mit Colchicin vorherzusagen [16].

Eine rezente Arbeit stellte fest, dass CYP1A1 womöglich ebenfalls am Metabolismus von Colchicin mitwirkt und dessen Hepatotoxizität intensiviert [23]. Die Hemmung von CYP1A1 konnte im Mausmodell oxidativen Stress und Pyroptose in der Leber nach einer Colchicin-Behandlung lindern, was das Enzym zu einem interessanten Ziel zur Vermeidung einer Colchicin -verursachten Leberschädigung macht [23]. Ein allfälliges Risiko für kognitive Beeinträchtigungen und Demenz betrifft laut einer großen Kohortenstudie nur Patienten mit Gichtdiagnose und einer höheren kumulativen Gesamtdosis (> 30) von Colchicin [260]. Inwiefern Gicht, Demenz und Colchicin kausal zusammenhängen, ist im Augenblick noch unbekannt.

Um die sichere Langzeitanwendung von Colchicin bei Patienten mit atherosklerotischer Erkrankung der Herzkranzgefäße zu gewährleisten, wurde eine aktuelle Literatursuche durchgeführt (vgl. Tab. 1). In dieser Tabelle werden verschiedene Enzyminhibitoren und deren Potenz, basierend auf der Erhöhung der AUC ausgewählter Substrate, dargestellt. Dazu gehören auch viele CYP3A4- und P‑gp-Inhibitoren, die häufig von Patienten mit atherosklerotischer Erkrankung der Herzkranzgefäße eingenommen werden. Aufgrund eines Mangels an Interaktionsdaten zwischen Colchicin und den angegebenen Inhibitoren wurden sog. „Probe-Drugs“ – Substrate mit ähnlichem Metabolismus wie Colchicin – herangezogen. Gemäß der EMA „Guideline on drug interaction studies“ (EMA/CHMP/ICH/652460/2022) wurde für die CYP3A4-Aktivität vorwiegend Midazolam und für die P‑gp-Aktivität primär Digoxin als Substrat ausgewählt. Falls keine pharmakokinetischen Studien zu Colchicin oder den Substraten Midazolam und Digoxin verfügbar waren, wurden alternative Substrate mit ähnlichem Metabolismus wie Colchicin herangezogen. Abweichungen in der Einstufung als schwacher, moderater oder starker CYP-Inhibitor können ggf. durch unterschiedliches Studiendesign, unterschiedliche Populationen oder variierende Dosierungen entstehen.

**Tab. 1:** *Interaktionspotenzial. *Colchicin kann mit anderen Arzneistoffen wechselwirken. Die Tabelle zeigt die Zuordnung in starke, moderate und schwache Interaktionen und gibt konkrete Empfehlungen, wie in der Praxis vorzugehen ist

Wirkstoff	Outcome	Anmerkung	Referenz
*Starke CYP3A4- und/oder P‑gp-Inhibitoren (erhöhen die AUC ≥* *5fach)*			
Atazanavir	Es wurde ein signifikanter Anstieg der Colchicin-Plasmaspiegel beobachtet Bei Clarithromycin, einem starken CYP3A4-Inhibitor, und Cyclosporin, einem starken P‑gp-Inhibitor, wurde über tödliche Colchicin-Toxizität berichtet Auch bei anderen starken CYP3A4- und P‑gp-Inhibitoren ist mit einem signifikanten Anstieg der Colchicin-Plasmaspiegel zu rechnen	Die gleichzeitige Anwendung von Colchicin mit starken CYP3A4- oder P‑gp-Inhibitoren ist kontraindiziert Colchicin soll 2 Tage vor Beginn einer Therapie mit Makrolidantibiotika pausiert werden	SmPC Colcardio®CONOVA 0,5 mg Tabletten
Clarithromycin			SmPC Colcardio®CONOVA 0,5 mg Tabletten
Cyclosporin			SmPC Colcardio®CONOVA 0,5 mg Tabletten
Darunavir/Ritonavir			SmPC Colcardio®CONOVA 0,5 mg Tabletten
Fluconazol			SmPC Colcardio®CONOVA 0,5 mg Tabletten
Indinavir			SmPC Colcardio®CONOVA 0,5 mg Tabletten
Itraconazol			SmPC Colcardio®CONOVA 0,5 mg Tabletten
Ketoconazol			SmPC Colcardio®CONOVA 0,5 mg Tabletten
Lopinavir/Ritonavir			SmPC Colcardio®CONOVA 0,5 mg Tabletten
Nefazodon			SmPC Colcardio®CONOVA 0,5 mg Tabletten
Nelfinavir			SmPC Colcardio®CONOVA 0,5 mg Tabletten
Nirmatrelvir/Ritonavir			SmPC Colcardio®CONOVA 0,5 mg Tabletten
Ranolazin			SmPC Colcardio®CONOVA 0,5 mg Tabletten
Ritonavir			SmPC Colcardio®CONOVA 0,5 mg Tabletten
Saquinavir			SmPC Colcardio®CONOVA 0,5 mg Tabletten
Telithromycin			SmPC Colcardio®CONOVA 0,5 mg Tabletten
Tipranavir/Ritonavir			SmPC Colcardio®CONOVA 0,5 mg Tabletten
Voriconazol			SmPC Colcardio®CONOVA 0,5 mg Tabletten
*Moderate CYP3A4- und/oder P‑gp-Inhibitoren (erhöhen die AUC ≥* *2- bis <* *5fach)*			
Amprenavir	Es wurde ein signifikanter Anstieg der Colchicin-Plasmakonzentration beobachtet Es gibt Berichte über neuromuskuläre Toxizität in Kombination mit Verapamil und Diltiazem	Nutzen-Risiko-Abwägung Patienten sorgfältig auf Anzeichen und Symptome einer Toxizität überwachen Die gemeinsame Anwendung von Colchicin mit moderaten CYP3A4- oder P‑gp-Inhibitoren bei älteren Patienten und bei Patienten mit eingeschränkter Nieren- oder Leberfunktion ist zu vermeiden	SmPC Colcardio®CONOVA 0,5 mg Tabletten
Aprepitant			SmPC Colcardio®CONOVA 0,5 mg Tabletten
Diltiazem			SmPC Colcardio®CONOVA 0,5 mg Tabletten
Dronedaron			SmPC Colcardio®CONOVA 0,5 mg Tabletten
Erythromycin			SmPC Colcardio®CONOVA 0,5 mg Tabletten
Fosamprenavir			SmPC Colcardio®CONOVA 0,5 mg Tabletten
Grapefruitsaft			SmPC Colcardio®CONOVA 0,5 mg Tabletten
Verapamil			SmPC Colcardio®CONOVA 0,5 mg Tabletten
*Schwache CYP3A4- und/oder P‑gp-Inhibitoren (erhöhen die AUC ≥* *1,25- bis <* *2fach)*			
Amiodaron	Pharmakokinetische und/oder pharmakodynamische Wechselwirkungen Cholesterin-Synthese-Hemmer können in Kombination mit Colchicin zu einem erhöhten Risiko von Myopathien und Rhabdomyolysen mit Nierenversagen führen Es kann hilfreich sein, auf Rosuvastatin oder Pravastatin auszuweichen, für die keine CYP3A4-Interaktionen beschrieben sind	Patienten auf Anzeichen und Symptome einer Toxizität überwachen Gegebenenfalls Colchicin-Dosis reduzieren oder Intervall verlängern, v. a. bei Patienten mit eingeschränkter Nierenfunktion (GFR < 30 ml/min)	10.1097/00005344-199610000-00009
Apixaban			10.1093/eurheartj/ehy136
Atorvastatin			SmPC Colcardio®CONOVA 0,5 mg Tabletten
Azithromycin			SmPC Zithromax 500 mg Filmtabletten
Cimetidin			10.1038/clpt.1987.13 10.1111/j.1600-0773.1986.tb00076.x
Clopidogrel			10.1111/jth.12445
Digoxin			SmPC Colcardio®CONOVA 0,5 mg Tabletten
Esomeprazol			10.1002/cpt.1949
Fibrate			SmPC Colcardio®CONOVA 0,5 mg Tabletten
Fluvastatin			SmPC Colcardio®CONOVA 0,5 mg Tabletten
Fluvoxamin			10.1177/0091270003259216 10.1007/BF00195913
Gemfibrozil			SmPC Colcardio®CONOVA 0,5 mg Tabletten
Phenprocoumon			SmPC MARCOUMAR – Tabletten 10.1111/bcpt.12084
Prasugrel			10.1111/jth.12445
Propafenon			PMID: 3732716
Rivaroxaban			10.1093/eurheartj/ehy136
Simvastatin			SmPC Colcardio®CONOVA 0,5 mg Tabletten
Ticagrelor			10.1093/eurheartj/ehy136
*Keine relevanten Interaktionen*			
Acenocoumarol	Keine relevanten Interaktionen	Keine Dosisanpassung von Colchicin notwendig	SmPC Sintrom 4 mg Tabletten
Acetylsalicylsäure			SmPC Thrombo ASS 100 mg Filmtabletten 10.1038/clpt.2014.49
Aminoglykoside			SmPC Amikacin B. Braun 5 mg/ml Infusionslösung SmPC Gentamicin B. Braun 3 mg/ml Infusionslösung SmPC Tobrasix 160 mg/2 ml Injektionslösung
Amlodipin			SmPC Norvasc 10 mg Tabletten
Atenolol			SmPC Tenormin 100 mg – Filmtabletten
Bempedoinsäure			SmPC Nilemdo 180 mg Filmtabletten
Bisoprolol			SmPC Bisocor 10 mg – Filmtabletten
Candesartan			SmPC Blopress 32 mg – Tabletten
Bumetanid			SmPC Burinex 1 mg – Tabletten
Carvedilol			SmPC Dilatrend 25 mg – Tabletten
Chinolone			SmPC Ciprobay 500 mg Filmtabletten SmPC Quofenix 450 mg Tabletten SmPC Tavanic 500 mg Filmtabletten SmPC Avalox 400 mg Filmtabletten SmPC Floxacin 400 mg Filmtabletten SmPC Ofloxacin Stada 400 mg Filmtabletten SmPC Unidrox 600 mg Filmtabletten
Chlortalidon			SmPC Hydrosan 25 mg Tabletten
Cilazapril			SmPC Inhibace 5 mg – Filmtabletten
Clindamycin			SmPC Dalacin C® 300 mg – Kapseln
Clonidin			SmPC Catapresan 0,15 mg – Tabletten
Dabigatran			SmPC Pradaxa 110/150 mg Hartkapseln 10.1093/eurheartj/ehy136
Dapagliflozin			SmPC Forxiga® 10 mg Filmtabletten
Diosmin			SmPC Daflon 500 mg – Filmtabletten
Doxazosin			SmPC Supressin 4 mg Tabletten
Doxycyclin			SmPC Vibramycin 200 mg – lösbare Tabletten
Dulaglutid			SmPC Trulicity 4,5 mg Injektionslösung
Edoxaban			SmPC 10.1093/eurheartj/ehy136
Empagliflozin			SmPC Jardiance 25 mg Filmtabletten
Enalapril			SmPC Mepril 20 mg – Tabletten
Enoxaparin			SmPC LOVENOX 4000 IE (40 mg)/0,4 ml Injektionslösung
Eplerenon			SmPC Inspra 50 mg Filmtabletten
Eprosartan			SmPC Teveten 600 mg – Filmtabletten
Ezetimib			SmPC Ezetimib + pharma 10 mg Tabletten
Felodipin			SmPC Plendil retard 5 mg – Filmtabletten
Flecainid			SmPC Aristocor 100 mg – Tabletten
Fosfomycin			SmPC Monuril 3 g Granulat
Fosinopril			SmPC Fositens 20 mg Tabletten
Furosemid			SmPC Lasix (alle in Österreich erhältlichen Darreichungsformen)
Gliclazid			SmPC Diamicron MR 60 mg Retard-Tabletten
Glimepirid			SmPC Glimepirid Hexal 4 mg Tabletten
Gliquidon			SmPC Glurenorm 30 mg Tabletten
Hydrochlorothiazid			SmPC HCT G.L. 50 mg Tabletten
Imidapril			SmPC Tanatril 20 mg Tabletten
Indapamid			SmPC Fludex 1,5 mg Filmtabletten
Insulin (Anm.: sämtliche in Österreich erhältlichen Darreichungsformen mit dem in der Spalte „Referenz“ genannten Markennamen)			SmPC Actraphane SmPC Actrapid SmPC Apidra SmPC Fiasp SmPC Humalog SmPC HumalogMix SmPC Huminsulin SmPC Insulatard SmPC Lantus SmPC Levemir SmPC Lyumjev SmPC Mixtard SmPC NovoMix SmPC NovoRapid SmPC Toujeo SmPC Tresiba
Irbesartan			SmPC Irbepress 150 mg Filmtabletten
Lercanidipin			SmPC Zanidip 20 mg Filmtabletten
Linagliptin			SmPC Trajenta 5 mg Filmtabletten
Liraglutid			SmPC Victoza 6 mg/ml Injektionslösung in Fertigpen SmPC Saxenda 6 mg/ml Injektionslösung in Fertigpen
Lisinopril			SmPC Acemin 20 mg Tabletten
Losartan			SmPC Losartan + pharma 100 mg Filmtabletten
Metformin			SmPC Glucophage 1000 mg Filmtabletten
Metoprolol			SmPC Beloc 100 mg Tabletten SmPC Beloc 100 mg Ampullen
Moxonidin			SmPC Moxonidin 0,4 mg Filmtabletten
Nebivolol			SmPC Nomexor 5 mg Tabletten
Penicilline und Cephalosporine (Anm.: sämtliche in Österreich erhältlichen Darreichungsformen mit dem in der Spalte „Referenz“ genannten Markennamen)			SmPC Augmentin SmPC Biocef SmPC Ceclor SmPC Ospen SmPC Ospexin SmPC Pipitaz SmPC Selexid SmPC Tricef SmPC Unasyn SmPC Zinnat
Pentoxifyllin			SmPC Trental 400 mg Filmtabletten
Pioglitazon			SmPC Actos 30 mg Tabletten
Pravastatin			SmPC Panchol 40 mg Tabletten
Propranolol			SmPC Inderal 40 mg – Filmtabletten
Ramipril			SmPC Tritace 5 mg Tabletten
Repaglinid			SmPC NovoNorm 2 mg Tabletten
Rilmenidin			SmPC Iterium 1 mg – Tabletten
Rosuvastatin			SmPC Crestor 40 mg Filmtabletten
Roxithromycin			SmPC Roxithromycin + pharma 300 mg Filmtabletten
Saxagliptin			SmPC Onglyza 5 mg Filmtabletten
Semaglutid			SmPC Ozempic 1 mg Injektionslösung in Fertigpen SmPC Wegovy 2,4 mg Injektionslösung in Fertigpen SmPC Rybelsus 14 mg Tabletten
Spironolacton			SmPC ALDACTONE 50 mg – überzogene Tabletten
Sitagliptin			SmPC Januvia 100 mg Filmtabletten
Sulfonamide ± Trimethoprim			SmPC Motrim 200 mg Tabletten SmPC Eusaprim forte Tabletten SmPC Lidaprim forte Filmtabletten
Telmisartan			SmPC Micardis 80 mg Tabletten
Torasemid			SmPC Torasemid Hexal 20 mg Tabletten
Urapidil			SmPC Uratens 60 mg Hartkapseln
Valsartan			SmPC Diovan 160 mg Filmtabletten
Vildagliptin			SmPC Galvus 50 mg Tabletten

*AUC* „area under the curve“, *P‑gp* P-Glykoprotein*, SmPC* „summary of product characteristics“

## Gicht

Gicht ist eine wiederkehrende, fortschreitende, entzündliche, systemische, muskuloskeletale Erkrankung, die durch Speicherung von Mononatriumurat-Kristallen (MSU) im mesenchymalen Gewebe verursacht wird [22]. MSU entstehen, wenn die Löslichkeitsgrenze von Natriumurat im Serum überschritten ist [22]. Diese liegt unter physiologischen pH- und Temperaturverhältnissen bei etwa 6,8 mg/dl [24]. Sind die Werte permanent erhöht, können sich auf Dauer Tophi bilden, die zu Erosionen und irreversibler Gelenkschädigung bzw. -destruktion führen [24]. Die Progression von einer asymptomatischen Hyperurikämie hin zu einer manifesten Gicht ist ein mehrstufiger Prozess, dessen genauer Hergang noch erforscht wird [25]. Fest steht, dass Gicht mit einer Prävalenz von 2–4 % die häufigste entzündliche Gelenkserkrankung der westlichen Welt darstellt und mit zahlreichen kardiovaskulären (Hypertonie, ACS, Herzinsuffizienz, Schlaganfall) und renalen (chronische Niereninsuffizienz, Nephrolithiasis) Komorbiditäten assoziiert ist [26–28]. Frauen sind diesbezüglich ab der Menopause stärker gefährdet als Männer, weil dann der protektive Effekt des Östrogens wegfällt [29]. Die Mortalität ist verglichen mit der Normalbevölkerung bei beiden Geschlechtern erhöht [30, 31].

Dies untermauern die Ergebnisse einer kürzlich im *Lancet Rheumatology* publizierten Fall-Kontroll-Studie, welche die Inzidenz von zwölf Herz-Kreislauf-Erkrankungen bei Patienten mit erstmalig diagnostizierter Gicht untersuchte [32]. Die Kontrollkohorte umfasste bis zu fünf Kontrollpersonen pro Patient mit Gicht, die nach Alter, Geschlecht, sozioökonomischem Status, geografischer Region und Kalenderzeit auf die Gicht-Gruppe abgestimmt waren [32]. Insgesamt wurden 152.663 Personen mit Gicht und 709.981 Kontrollpersonen identifiziert von denen 31.479 (20,6 %) Patienten mit Gicht und 106.520 (15,0 %) Personen ohne Gicht während einer mittleren Nachbeobachtungszeit von 6,5 Jahren eine Herz-Kreislauf-Erkrankung entwickelten (Hazard Ratio [HR] 1,58 [95 %-KI 1,52–1,63]) [32]. Das Risiko blieb auch nach Anpassung für kardiovaskuläre Risikofaktoren erhöht (bereinigte HR 1,31 [1,27–1,36]) [32]. Frauen und jüngeren Personen unter 45 Jahren waren am stärksten gefährdet [32].

**Abb. 1:**
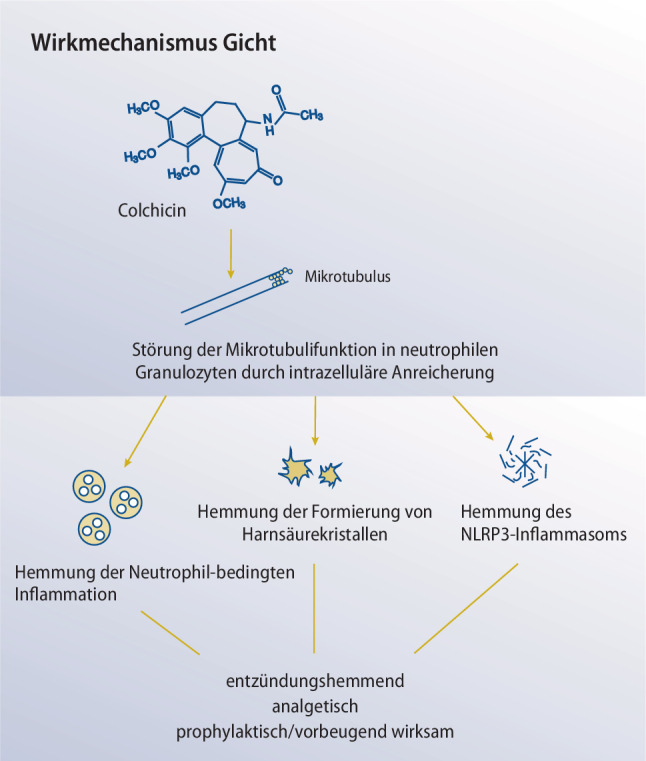
*Wirkungsmechanismus bei Gicht. *Die Wirksamkeit von Colchicin bei Gicht beruht primär auf seiner Fähigkeit sich in neutrophilen Granulozyten anzureichern und deren Mikrotubulifunktion zu stören. Dies hemmt die Bildung des NLRP3-Inflammasoms, unterdrückt die neutrophile Entzündungsreaktion und verhindert die Formierung von Harnsäurekristallen

Der Stellenwert von Colchicin zur Behandlung der Gicht ist in allen internationalen Empfehlungen verankert [22, 33–37]. Gemäß den aktuellen European Alliance of Associations for Rheumatology(EULAR)-Leitlinien ist es ein Mittel der ersten Wahl zur Anfallskupierung und in der Anfallsprophylaxe [34]. Aufgrund seiner vielfältigen Wirkmechanismen werden durch Colchicin verschiedene pathogenetische Aspekte der Gicht positiv beeinflusst [38]. Dazu zählen die Hemmung der Polymerisierung von Mikrotubuli in neutrophilen Granulozyten, die Hemmung der Adhäsion, Extravasation und Rekrutierung von Neutrophilen, die Modulation der Leukozyten-bedingten Inflammation sowie die Hemmung der Caspase-1-, IL-1β- und IL-18-Aktivierung [38]. Letzteres dürfte auf der Fähigkeit von Colchicin beruhen, die MSU-induzierte Aktivierung des Nod-Like-Receptor-Protein 3(NLRP3)-Inflammasoms zu unterdrücken (vgl. Abb. 1; [39]).

NLRP3 ist für die Bildung der Caspase‑1 zuständig, die Pro-IL-1β zu IL-1β spaltet, was die Freisetzung nachgeschalteter proinflammatorischer Zytokine wie IL‑6 und IL-18 fördert [39]. Auf diese Weise werden neutrophile Granulozyten rekrutiert, welche die lokale Entzündung unterhalten und verstärken [39]. Diese Beobachtungen stimmen damit überein, dass mit Colchicin vorwiegend Erkrankungen behandelt werden, bei denen Neutrophile und Monozyten/Makrophagen – sprich Zellen des angeborenen Immunsystems – beteiligt sind [38]. Ermöglicht wird dies durch die hohe intrazelluläre Colchicin-Konzentration in Neutrophilen [40]. Diese kann mehr als das 16fache der Spitzenkonzentration im Plasma betragen [41]. Der genaue Mechanismus, durch den Colchicin das NLRP3-Inflammasom hemmt, ist bis dato unbekannt [42]. Er könnte aber mit der Störung des Mikrotubuli-abhängigen Transports von Mitochondrien zum endoplasmatischen Retikulum zusammenhängen, wodurch Colchicin die Schwelle zur Auslösung einer vollständigen NLRP3-Inflammasom-Aktivierung erhöht [43]. Marques-da-Silva et al. konnten im Mausmodell demonstrieren, dass Colchicin ein wirksamer Inhibitor der P2X7-Porenbildung ist [44]. Die Bildung von P2X7-Poren ist ein notwendiger Schritt der angeborenen Immunantwort, um NLRP3 zu aktiveren [44]. In Summe wirkt Colchicin bei Gicht entzündungshemmend, analgetisch und hat eine inhibierende Wirkung auf die Ablagerung von Harnsäurekristallen [16].

### Anfallskupierung

Für den akuten Gichtanfall empfehlen sämtliche Guidelines gleichwertig Colchicin, Glukokortikoide und nichtsteroidale antiinflammatorische Arzneistoffe (NSAR) [22, 33–37]. Die medikamentöse Anfallskupierung sollte so schnell wie möglich (innerhalb von 12 h nach Anfallsbeginn) als Selbstmedikation durch den Patienten selbst erfolgen („pill in the pocket“) [22, 33–37]. Die früher übliche Hochdosistherapie mit Colchicin mit einer Initialdosis von 1,2 mg gefolgt von einer stündlichen Gabe von 0,6 mg für 6 h bzw. bis zum Eintritt von Nebenwirkungen ist seit der Arbeit von Terkeltaub et al. obsolet [45]. In ihrer doppelblinden, placebokontrollierten Studie wurden 575 Patienten mit akuter Gicht entweder auf eine Hochdosistherapie mit Colchicin, ein Niedrigdosisschema (initial 1,2 mg, gefolgt von 1‑malig 0,6 mg nach 1 h) oder Placebo randomisiert [45]. Den Patienten wurde gesagt, dass sie ihre Medikamente innerhalb von 12 h nach Einsetzen der Symptome einnehmen sollen [45]. Es berichteten 37,8 % in der Hochdosis- gegenüber 32,7 % in der Niedrigdosis- und 15,5 % in der Placebogruppe über eine 50 %ige Besserung der Schmerzen [45]. Allerdings kam es bei 19,2 % der Patienten in der Hochdosisgruppe zu Durchfall, wohingegen in der Niedrigdosisgruppe die Nebenwirkungsrate sich nicht signifikant von Placebo unterschied [45]. Das Niedrigdosisschema (länderspezifisch initial 1,2 mg oder 1 mg gefolgt von 0,6 mg oder 0,5 mg nach 1 h) wurde daraufhin in alle Empfehlungen übernommen und kann bei Bedarf auch mit NSAR oder Glukokortikoiden kombiniert werden [22, 33–37]. Nach Beginn der Akutbehandlung ist eine Folgetherapie mit 0,5 mg Colchicin 1‑ oder 2‑mal täglich beginnend 12 h später bis zum Abklingen der Gichtsymptome zu erwägen [34].

### Anfallsprophylaxe

Bei Patienten, die mit einer harnsäuresenkenden Therapie (ULT) beginnen, besteht über einen längeren Zeitraum ein erhöhtes Risiko für akute Gichtanfälle, was vermutlich mit der Mobilisierung abgelagerter Kristalle während der Auflösung fester MSU zusammenhängt [46]. Dementsprechend empfiehlt die EULAR eine längere tägliche Behandlung mit einem entzündungshemmenden Mittel, bis der Höhepunkt des Risikos für einen Gichtanfall abgeklungen ist [34]. Die tatsächliche Dauer soll individuell auf den Patienten, die Komorbiditäten, die Komedikation und die Schwere der Erkrankung abgestimmt werden und beträgt 8 Wochen bis 6 Monate [34]. Colchicin (0,5–1 mg/Tag) wird als Mittel der ersten Wahl genannt, insbesondere bei komorbider entzündlicher gastrointestinaler Erkrankung, zurückliegendem Ulkus oder erhöhtem kardiovaskulärem Risiko [34]. Diese Erkenntnisse stammen einerseits aus der Studie von Borstad et al., welche die Wirksamkeit der gleichzeitigen Anwendung von Colchicin mit Allopurinol im 6‑monatigen Studienzeitraum zur Anfallsprophylaxe bewertete [46]; 33 % der Patienten unter Colchicin vs. 77 % der Patienten unter Placebo erlitten mindestens einen Gichtanfall [46]. Stattgefundene Anfälle wurden von den Patienten in der Colchicin-Gruppe weniger schwer empfunden als von denjenigen in der Placebogruppe [46]. Eindrucksvoll ist auch die Number Needed to Treat (NNT) von 2 [46]. Das heißt, es müssen 2 Patienten über 6 Monate begleitend mit Colchicin behandelt werden, um einen Gichtanfall unter laufender Allopurinol-Therapie zu verhindern [46]. Andererseits war Colchicin als Prophylaxe auch in den Zulassungsstudien von Febuxostat erlaubt [47–49]. Niedrig dosierte NSAR oder Glukokortikoide sind nur dann indiziert, wenn Colchicin nicht toleriert wird oder kontraindiziert ist [34]. Die Tab. 2 gibt einen Überblick über die einzelnen Studien und Leitlinien, die sich mit Colchicin zur Gichttherapie beschäftigen.

**Tab. 2:** *Studiendaten Gicht. *Auflistung von Studien, die den Einsatz von Colchicin bei Gicht untersucht haben unter Berücksichtigung der klinischen Situation, eingesetzten Dosierung, Anwendungsdauer, des Outcomes sowie der häufigsten Nebenwirkung

Studien	Klinische Situation	Dosierung	Anwendungsdauer	Ergebnis	Häufigste UAW unter Colchicin
Terkeltaub et al. [45]	Akuter Gichtanfall	1,2 mg Colchicin gefolgt von stündlich 0,6 mg Colchicin für 6 h (Hochdosis) oder 1,2 mg Colchicin gefolgt von 1‑malig 0,6 mg nach 1 h (Niedrigdosis) oder Placebo	1‑malig, innerhalb von 12 h	37,8 % in der Hochdosis- vs. 32,7 % in der Niedrigdosis- und 15,5 % in der Placebogruppe berichteten über eine 50 %ige Besserung der Schmerzen	76,9 % Durchfall in der Hochdosisgruppe vs. 23,0 % in der Niedrigdosisgruppe vs. 13,6 % mit Placebo
Borstad et al. [46]	Gichtprophylaxe	Allopurinol plus 0,6 mg Colchicin 2‑mal/Tag oder Placebo	6 Monate	33 % unter Colchicin vs. 77 % unter Placebo erlitten ≥ 1 Gichtanfall	Durchfall bei 38,5 % vs. 4,5 % mit Placebo
Sautner et al. [22]	Österreichische Leitlinie	Akut: 1 mg Colchicin möglichst rasch, gefolgt von 1‑malig 0,5 mg Colchicin nach 1 h Prophylaxe: 0,5 mg Colchicin 1‑ bis 2‑mal täglich	Akut: 1‑malig, möglichst rasch nach Anfallsbeginn Prophylaxe: 3 bis 6 Monate, begleitend zur harnsäuresenkenden Therapie	First-Line für Akuttherapie und Prophylaxe	–
Kiltz et al. [33]	Deutsche Leitlinie	Akut: 0,5–1,5 mg Colchicin, je nach Schwere des Anfalls und Komorbidität Prophylaxe: 0,5 mg Colchicin 2‑mal täglich	Akut: 1‑malig, möglichst rasch nach Anfallsbeginn Prophylaxe: 3 bis 6 Monate, begleitend zur harnsäuresenkenden Therapie	First-Line für Akuttherapie und Prophylaxe	–
Richette et al. [34]	Europäische EULAR-Leitlinie	Akut: 1 mg Colchicin innerhalb von 12 h nach Anfallsbeginn, gefolgt von 1‑malig 0,5 mg Colchicin nach 1 h Prophylaxe: 0,5 mg Colchicin 1‑ bis 2‑mal täglich	Akut: 1‑malig, innerhalb von 12 h Prophylaxe: 6 Monate, begleitend zur harnsäuresenkenden Therapie	First-Line für Akuttherapie und Prophylaxe	–
FitzGerald et al. [35]	Amerikanische ACR-Leitlinie	Akut: 1,2 mg Colchicin innerhalb von 12 h nach Anfallsbeginn, gefolgt von 1‑malig 0,6 mg Colchicin nach 1 h Prophylaxe: 0,6 mg Colchicin 1‑ bis 2‑mal täglich	Akut: 1‑malig, innerhalb von 12 h Prophylaxe: 6 Monate, begleitend zur harnsäuresenkenden Therapie	First-Line für Akuttherapie und Prophylaxe	–
Hisatome et al. [36]	Japanische Leitlinien	Akut: 1 mg Colchicin innerhalb von 12 h nach Anfallsbeginn, gefolgt von 1‑malig 0,5 mg Colchicin nach 1 h Prophylaxe: 0,5 mg Colchicin 1‑ bis 2‑mal täglich	Akut: 1‑malig, innerhalb von 12 h Prophylaxe: 6 Monate, begleitend zur harnsäuresenkenden Therapie	First-Line für Akuttherapie und Prophylaxe	–
Hui et al. [37]	Britische Leitlinien	Akut: 1 mg Colchicin innerhalb von 12 h nach Anfallsbeginn, gefolgt von 1‑malig 0,5 mg Colchicin nach 1 h Prophylaxe: 0,5 mg Colchicin 1‑ bis 2‑mal täglich	Akut: 1‑malig, möglichst rasch nach Anfallsbeginn Prophylaxe: 6 Monate, begleitend zur harnsäuresenkenden Therapie	First-Line für Akuttherapie und Prophylaxe	–

*ACR* American College of Rheumatology, *EULAR* European Alliance of Associations for Rheumatology, *UAW* unerwünschte Arzneimittelwirkung

## Familiäres Mittelmeerfieber

FMF ist eine autosomal-rezessiv vererbte, autoinflammatorische Erkrankung, die gehäuft bei Bevölkerungsgruppen der östlichen Mittelmeerregion auftritt [50]. Hierzu zählen in erster Linie Armenier, Türken, Muslime aus dem Nahen Osten, Bewohner von Nordafrika und nicht-aschkenasische Juden [51]. Die FMF-Prävalenz bei diesen Menschen wird generell mit 1:200 bis 1:1000 angegeben [51]. Durch Migrationsbewegungen im Laufe der Jahrhunderte hat sich die Erkrankung auch in Europa ausgebreitet, wobei südlich gelegene Länder wie Italien, Frankreich und Griechenland angesichts ihrer geografischen Nähe zu den Ursprungsländern die höchsten Inzidenzraten verzeichnen [52].

Charakteristisch für FMF sind wiederkehrende, akute, selbstlimitierende Fieberschübe mit begleitenden abdominellen Beschwerden, die durchschnittlich 24–72 h andauern [50]. Der entzündliche Ausbruch betrifft seröse Membranen (Serositiden), sodass FMF Symptome einer Peritonitis, Pleuritis, Perikarditis, Synovitis und/oder Orchitis verursachen kann [49]. Ursächlich ist eine Genmutation des *MEFV*-Gens (MEditerrean FeVer), welches für Pyrin kodiert, ein Protein, das an der Regulation von Entzündungen beteiligt ist [53]. MEFV wird hauptsächlich in Granulozyten, Monozyten, dendritischen Zellen sowie serösen und synovialen Fibroblasten exprimiert [54]. Es ist an der Aktivierung von Caspase‑1 und damit indirekt an der Freisetzung proinflammatorischer Zytokine beteiligt [54].

Die schwerwiegendste Komplikation von FMF ist die systemische Amyloidose, die v. a. die Nieren in Mitleidenschaft zieht und ein chronisches Nierenversagen mit Transplantationsbedarf begünstigt [51]. Sie entwickelt sich je nach Genotyp und Abstammung bei einem kleinen Prozentsatz der Patienten, besonders bei zugrunde liegender M694V-Mutation, seltener bei E148Q, V726A, A744S und R202Q [52, 69]. Amyloidose bei FMF ist gekennzeichnet durch die Polymerisation von Serumamyloid A zu Amyloidfibrillen und deren Ablagerung in mehreren Organen [55]. Neben den Nieren sind dies oft das Herz, die Leber, Milz und Hoden [55].

Ziel der Therapie ist es, Anfälle zu verhindern, die subklinische Entzündung im Intervall weitestgehend zu reduzieren und die Entstehung bzw. Progression einer Amyloidose zu vermeiden [56]. Mittel der Wahl, um diese Ziele zu erreichen, ist Colchicin [56]. Es eignet sich sowohl zur Akuttherapie als auch zur Vorbeugung von Amyloidose sowie der damit einhergehenden Verschlechterung der Nierenfunktion und Proteinurie und wird seit mehr als 40 Jahren erfolgreich zur Behandlung des FMF eingesetzt [56]. Der Wirkungsmechanismus ist dualer Natur: Colchicin reduziert indirekt den Serumamyloid-A-Spiegel und blockiert direkt die Ablagerung von Amyloidfibrillen [55]. Überdies stört es die Aktivierung, Degranulation und Migration von neutrophilen Granulozyten, indem es die Bildung mitotischer Spindeln hemmt [57]. Colchicin bindet dafür an die Domäne C der β‑Untereinheit, ändert ihre Konformation und unterbindet so, dass weitere Dimere in Längs- und Querrichtung hinzugefügt werden [57].

Die entzündungshemmenden Eigenschaften von Colchicin bei FMF ergeben sich aus der Synthesehemmung des Tumornekrosefaktors α (TNF-α), was konsekutiv die Oberflächenexpression des TNF-α-Rezeptors auf Makrophagen und Endothelzellen herunterreguliert und die P‑, E‑ und L‑Selektin-vermittelte endotheliale Anheftung von Neutrophilen blockiert [58, 59]. Auch der prophylaktische Effekt von Colchicin könnte auf diesen Mechanismus zurückzuführen sein. Andere Arbeiten berichten von einer Hemmung von Superoxidanionen, Phospholipase A2 und Lipoxygenase‑5, die vermutlich zur positiven Gesamtwirkung beitragen [60–62]. Das gilt auch für die hemmende Wirkung auf den intrazellulären Aufbau des NLRP3-Inflammasom-Komplexes in Neutrophilen und Monozyten sowie die nachfolgend verminderte Freisetzung von IL-1β [63].

### Empfehlungen zur Therapie

Die EULAR hat in ihrer Leitlinie zum Management des FMF 18 Empfehlungen für die Praxis erarbeitet [56]. Viele davon beziehen sich auf den korrekten Einsatz von Colchicin [56]. So soll die Therapie umgehend beginnen, sobald die Diagnose gestellt wurde, um akute fieberhafte Schmerzattacken sowie die Entwicklung und das Fortschreiten einer sekundären Amyloidose zu verhindern [56]. Die Tagesdosis kann man auf einmal einnehmen oder auf eine morgendliche und abendliche Gabe splitten [16, 56]. Erwachsene erhalten 1 mg bis maximal 3 mg pro Tag, Kinder 0,5 mg bis maximal 2 mg [16, 56]. Therapieerfolg, Verträglichkeit und Adhärenz sollten alle 6 Monate geprüft werden [57]. Die Behandlung erfolgt lebenslang und sollte auch während der Konzeption, Schwangerschaft und Stillzeit nicht unterbrochen werden [56]. Daten zu schwangeren Frauen mit FMF deuten auf kein Fehlbildungsrisiko oder fetale bzw. neonatale Toxizität unter Colchicin hin [16]. Stillen unter Colchicin ist erlaubt [16, 64]. Bislang existieren keine Hinweise für chromosomale Anomalien bei Männern, weshalb diese das Alkaloid vor dem Kinderzeugen nicht absetzen müssen [56]. Lediglich bei einer seltenen Azoo- oder Oligospermie wird eine Dosisreduktion oder Pausierung der Colchicin-Therapie empfohlen [56]. Wenn Patienten mehr als 5 Jahre schubfrei sind, einschließlich negativer Akute-Phase-Proteine, kann eine Reduktion der Colchicin-Dosis erwogen werden [56].

Die Evidenz für diese Therapieempfehlungen stammt aus mehreren Publikationen. Goldfinger (1972) war der Erste, der über die positive Wirkung von Colchicin bei FMF im *New England Journal of Medicine* berichtete, als er Patienten mit komorbider Gicht behandelte [65]. Sein Erfahrungsbericht legte den Grundstein der evidenzbasierten Colchicin-Therapie zur Vorbeugung von FMF-Anfällen und zur Verringerung des Amyloidoserisikos. Im Jahr 1974 folgte die erste doppelblinde, placebokontrollierte Studie, in der Patienten mit langjährigem FMF täglich Colchicin oder Placebo erhielten [66]. Während 60 Placebozyklen kam es zu 38 FMF-Anfällen, wohingegen in 60 Colchicin-Zyklen nur 7 Anfälle verzeichnet wurden [66]. Zwei weitere doppelblind-placebokontrollierte Studien bestätigen diese Ergebnisse [67, 68]. Im 4‑monatigen Crossover-Trial von Zemer et al. erhielten Teilnehmende 0,5 mg Colchicin oder Placebo 2‑mal täglich über 2 Monate, gefolgt von 2 Monaten der jeweils anderen Behandlung [67]. Bei Patienten in der Colchicin-Gruppe war die Anfallsrate deutlich niedriger (median 1,15 pro Patient) als in der Placebogruppe (median 5,25 pro Patient) [67]. Ben-Chetrit und Levy stellten ebenfalls eine vorbeugende Wirkung von Colchicin auf FMF- und Amyloidoseschübe fest [68]. Die tägliche Gabe führte zu einer signifikanten Reduktion der FMF-Anfallsfrequenz und des Risikos für Nierenversagen im Rahmen einer Amyloidose [68].

## Perikarditis

Der Herzbeutel (Perikard) ist ein doppelwandiger Beutel, der das Herz und die Wurzeln der großen Gefäße enthält [70]. Er besteht aus einer serösen viszeralen Schicht (Epikard) und einer faserigen Parietalschicht [70]. Das Perikard umschließt die Herzbeutelhöhle, fixiert das Herz am Mediastinum und schützt es vor Infektionen [70]. Entzündungen des Herzbeutels lassen sich grob in akute und rezidivierende Formen unterteilen [70]. Die akute Perikarditis beruht vielfach auf einer viralen Infektion und stellt die häufigste entzündliche Herzerkrankung dar [71]. Bei den Bakterien fungiert in der Regel *Staphylococcus aureus* als Auslöser [71]. Bedingt durch die reiche Innervation des Herzbeutels kann eine Perikarditis mit behindernden oder wiederkehrenden Brustschmerzen einhergehen, die sich beim Vorbeugen bessern [71]. Im Elektrokardiogramm zeigt sich üblicherweise eine ausgedehnte ST-Streckenhebung, im Echokardiogramm eventuell ein Perikarderguss [71]. Bei bis zu einem Drittel der Patienten ist auskultatorisch ein perikardiales Reiben im Sinne eines rauen, kratzenden oder schabenden Herzgeräusches vernehmbar, das im Verlauf meist leiser wird oder gänzlich verschwindet [70]. Erhöhte CRP-Werte im akuten Krankheitsstadium gelten als Indikator für ein erhöhtes Rückfallrisiko [72].

Eine rezidivierende Perikarditis ist definiert als das Wiederauftreten der für eine Perikarditis typischen Symptome und Anzeichen nach einer krankheitsfreien Zeit von mindestens 4 bis 6 Wochen bei Personen mit einer früheren Episode einer akuten Erkrankung [70]. Die Inzidenz liegt zwischen 15 und 30 % [70]. Man nimmt an, dass rezidivierende Perikarditiden auf autoinflammatorischen Prozessen beruhen, die durch eine unangemessene Aktivierung der angeborenen Immunabwehr gekennzeichnet sind [73]. IL‑1 scheint dabei eine Schlüsselrolle einzunehmen [73]. Diese Hypothese wird durch den Nachweis unspezifischer Autoantikörper und dem guten Ansprechen auf entzündungshemmende Substanzen wie NSAR und Colchicin bestärkt [74].

**Abb. 2:**
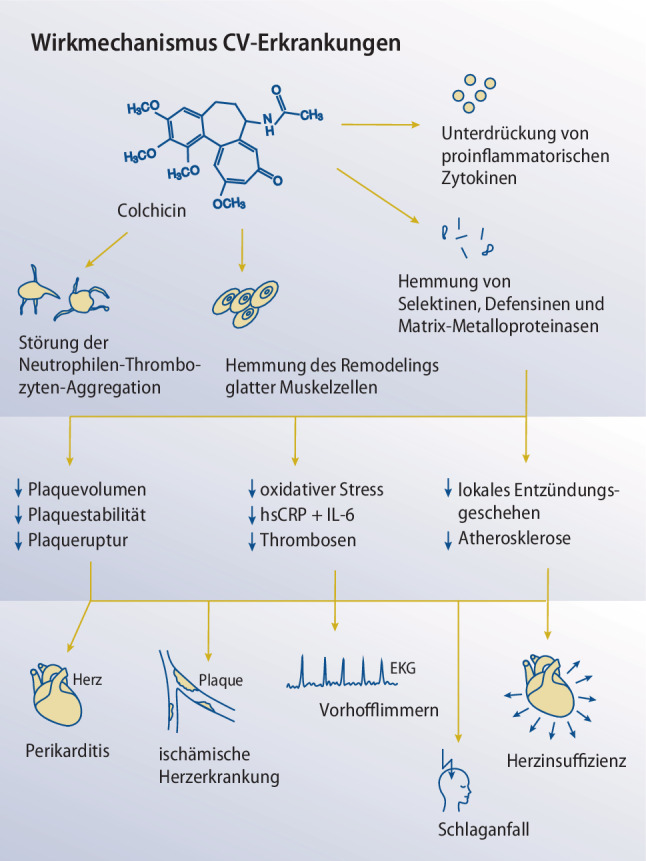
*Wirkungsmechanismus bei kardiovaskulären Erkrankungen. *Colchicin greift an mehreren Stellen korrigierend in das atherosklerotische Geschehen ein. Unter anderem reduziert es die endotheliale Expression von Selektinen und proinflammatorischen Zytokinen, die mit der Adhäsion und dem Eintritt von Leukozyten sowie der Proliferation glatter Gefäßmuskelzellen in Verbindung stehen. Colchicin inhibiert die Aktivierung neutrophiler Granulozyten, indem es sich intrazellulär anreichert und den Aufbau von Mikrotubuli im Zytoskelett verhindert. Die ausbleibende Interaktion zwischen Neutrophilen und Thrombozyten ist mit einer erhöhten Plaquestabilität und einem verringerten Plaquevolumen assoziiert. Die Summe dieser Einflüsse kann therapeutische Effekte bei verschiedenen kardiovaskulären Erkrankungen haben

Lange Zeit war nicht klar, auf welchen pathophysiologischen Mechanismen Perikarditiden basieren [75]. Das Ansprechen von Patienten mit akuter oder rezidivierender Perikarditis auf Colchicin spricht jedenfalls für eine Beteiligung neutrophiler Granulozyten, da Colchicin deren Migration und Phagozytose stört und modulierend in den Entzündungszyklus eingreift (vgl. Abb. 2; [75]). Positive Ergebnisse mit selektiven IL-1-Rezeptorantagonisten zur Behandlung der rezidivierenden idiopathischen Perikarditis haben ergeben, dass auch das NLRP3-Inflammasom an der Krankheitsentstehung beteiligt ist [76]. Die initiale Schädigung der perikardialen Mesothelzellen dürfte eine Entzündungsreaktion anstoßen, die durch die Aktivierung des NLRP3-Inflammasoms verstärkt und unterhalten wird [77, 78]. In Proben von Patienten mit chronischer Perikarditis war eine Aktivierung des NLRP3-Inflammasoms nachweisbar, wenn diese eine akute Episode bekamen [79]. Colchicin schiebt dem einen Riegel vor, indem es die korrekte Mikrotubuli-Zusammensetzung des Inflammasoms verhindert. Dieser Wirkmechanismus wurde bereits für Gicht-assoziierte MSU-Kristalle beschrieben [39].

Die Effektivität und Sicherheit von Colchicin bei akuter und rezidivierender Perikarditis wurde mittlerweile in mehreren randomisierten Studien nachgewiesen [80–85]. Hierfür wählte man eine Aufsättigungsdosis von 1–2 mg Colchicin am ersten Tag, gefolgt von täglich 0,5–1 mg (gewichtsadaptiert) für eine sich anschließende Erhaltungstherapie mit variabler Dauer (3 bis 6 Monate), oder man verzichtete auf die Initialdosis und begann gleich mit der Erhaltungsdosis [80–85]. Die Resultate waren, bis auf die kleine offene Studie von Sambola et al. (2019) bei Patienten mit akuter idiopathischer Perikarditis, positiv [80–85]. In der offen randomisierten COPE-Studie wurde Colchicin bei akuter Perikarditis ergänzend zur antiinflammatorischen Basistherapie untersucht [80]. Die Patienten erhielten dafür Acetylsalicylsäure (ASS) oder eine Initialdosis Colchicin am ersten Tag, gefolgt von einer Erhaltungsdosis für 3 Monate [80]. Colchicin reduzierte die Symptompersistenz nach 72 h (36,7 % vs. 11,7 %) sowie das Wiederauftreten der Perikarditis (10,7 % vs. 32,3 %) effektiver als ASS [80]. Die doppelblind-randomisierte ICAP-Studie war vergleichbar aufgebaut, allerdings ohne Initialdosis und mit Placebo anstelle von ASS [81]. Auch hier erwies sich Colchicin hinsichtlich der Symptompersistenz nach 72 h (40,0 % vs. 19,2 %), der Hospitalisierungsrate (5,0 % vs. 14,2 %), der Anzahl der Rezidive pro Patient (0,21 vs. 0,52) und der Remissionsrate nach einer Woche (85,0 % vs. 58,3 %) überlegen [81].

CORE und CORP (jeweils erstes Perikarditisrezidiv) sowie CORP‑2 (zweites oder darauffolgendes Rezidiv) prüften Colchicin bei rezidivierender Perikarditis [83–85]. Für CORE wurde eine Initialdosis Colchicin am ersten Tag, gefolgt von einer Erhaltungsdosis für 6 Monate gegen ASS als aktiven Komparator festgelegt [83]. Colchicin verminderte die Rezidivrate und Symptompersistenz nach 72 h signifikant stärker als ASS [83]. Die Ergebnisse konnten in den randomisiert kontrollierten CORP- und ICAP-Studien ohne Initialdosis bekräftigt werden, wo Colchicin gegenüber Placebo die Rezidivrate um 55 % vs. 24 % bzw. 42,5 % vs. 21,6 % reduzierte [84, 85]. Jüngste Daten signalisieren im Übrigen, dass Colchicin nach einer hybriden Sinusknoten-erhaltenden Ablation bei inadäquater Sinustachykardie bzw. posturalem orthostatischem Tachykardiesyndrom ebenso prophylaktisch wirksam ist und Perikarditiden vorbeugt [133]. Der Nutzen von Colchicin bei Perikarditis wird durch einen Cochrane-Review sowie die Empfehlung zur Erstbehandlung (Klasse I, Evidenzgrad A) als kosteneffizientes Add-on zur herkömmlichen antiinflammatorischen Therapie (ASS oder NSAR) bei Patienten mit erstmalig auftretender oder rezidivierender Perikarditis durch die Leitlinien der Europäischen Gesellschaft für Kardiologie (ESC) untermauert [70, 87]. Die Tab. 3 fasst die Studiendaten von Colchicin zur Perikarditistherapie zusammen.

**Tab. 3:** *Studiendaten Perikarditis. *Auflistung von Studien, die den Einsatz von Colchicin bei akuter und rezidivierender Perikarditis untersucht haben unter Berücksichtigung der klinischen Situation, eingesetzten Dosierung, Anwendungsdauer, des Outcomes sowie der häufigsten Nebenwirkung

Studien	Klinische Situation	Dosierung	Anwendungsdauer	Ergebnis	Häufigste UAW unter Colchicin
Imazio et al. [80]	Akute Perikarditis	1 mg Colchicin an Tag 1 gefolgt von 0,5 mg Colchicin/Tag (wenn < 70 kg) oder 1 mg Colchicin 2‑mal/Tag an Tag 1 gefolgt von 0,5 mg 2‑mal/Tag (wenn ≥ 70 kg)	3 Monate	Reduktion der Rezidivrate um 32,3 % mit Colchicin vs. 10,7 % ohne Colchicin Symptomreduktion nach 72 h um 36,7 % mit Colchicin vs. 11,7 % ohne Colchicin	Durchfall 8,3 % vs. 6,7 % ohne Colchicin
Imazio et al. [81]	Akute Perikarditis	0,5 mg Colchicin täglich (wenn < 70 kg) oder 0,5 mg Colchicin 2‑mal/Tag (wenn ≥ 70 kg)	3 Monate	Reduktion der rezidivierenden oder permanenten Perikarditis um 37 % mit Colchicin vs. 17 % ohne Colchicin Symptomreduktion nach 72 h um 40,0 % mit Colchicin vs. 19,2 % ohne Colchicin	Gastrointestinale Intoleranz bei 9,2 % vs. 8,3 % ohne Colchicin
Sambola et al. [82]	Akute Perikarditis	0,5 mg Colchicin 2‑mal/Tag (wenn < 70 kg) oder 1,0 mg Colchicin 2‑mal/Tag (wenn ≥ 70 kg)	3 Monate	Kein Unterschied zwischen Colchicin und ASS bzw. NSAID (13 % vs. 8 %) hinsichtlich Reduktion der rezidivierenden Perikarditis	Durchfall 13,5 % vs. 8,5 % ohne Colchicin
Imazio et al. [83]	Erstes Perikarditisrezidiv	1 mg Colchicin an Tag 1 gefolgt von 0,5 mg Colchicin/Tag (wenn < 70 kg) oder 2‑mal 1 mg Colchicin an Tag 1 gefolgt von 0,5 mg 2‑mal/Tag (wenn ≥ 70 kg)	6 Monate	Reduktion der rezidivierenden Perikarditis um 51 % mit Colchicin vs. 24 % ohne Colchicin Symptomreduktion nach 72 h um 31 % mit Colchicin vs. 10 % ohne Colchicin	Durchfall 7 % vs. 14 % ohne Colchicin
Imazio et al. [84]	Erstes Perikarditisrezidiv	1 mg Colchicin an Tag 1 gefolgt von 0,5 mg Colchicin/Tag (wenn < 70 kg) oder 2‑mal 1 mg Colchicin an Tag 1 gefolgt von 0,5 mg 2‑mal/Tag (wenn ≥ 70 kg)	6 Monate	Reduktion der rezidivierenden Perikarditis um 55 % mit Colchicin vs. 24 % ohne Colchicin Symptomreduktion nach 72 h um 53 % mit Colchicin vs. 23 % ohne Colchicin	Gastrointestinale Intoleranz 7 % vs. 5 % ohne Colchicin
Imazio et al. [85]	≥ 2 Perikarditisrezidive	0,5 mg Colchicin/Tag (wenn < 70 kg) oder 0,5 mg Colchicin 2‑mal/Tag (wenn ≥ 70 kg)	6 Monate	Reduktion der rezidivierenden Perikarditis um 42 % mit Colchicin vs. 22 % ohne Colchicin	Gastrointestinale Intoleranz 7,5 % vs. 7,5 % ohne Colchicin
Mohanty et al. [262]	Perikarditisinzidenz nach VHF-Ablation	Gruppe 1: kein Colchicin Gruppe 2: 0,3 mg Colchicin 2‑mal/Tag beginnend 7 Tage präoperativ Gruppe 3: 0,3 mg Colchicin 2‑mal/Tag beginnend am Tag der Ablation	1 Monat	Symptome einer akuten Perikarditis bei 17,5 % in Gruppe 1 vs. 1,9 % in Gruppe 2 vs. 7,5 % in Gruppe 3 Arrhythmiefreies Überleben im 1‑Jahres-Follow-up bei Patienten mit paroxysmalen VHF in Gruppe 2 und Gruppe 3 signifikant höher als in Gruppe 1	Gastrointestinale Intoleranz bei 8,5 % in Gruppe 1 vs. 4,3 % in Gruppe 2 vs. 4,3 % in Gruppe 3
Ahmed et al. [263]	Perikarditisinzidenz nach VHF-Ablation	0,6 mg Colchicin 2‑mal/Tag oder Standard-of-Care, beginnend unmittelbar nach der Ablation	7 Tage	Symptome einer akuten Perikarditis bei 9,6 % mit Colchicin vs. 10,6 % ohne Colchicin	Gastrointestinale Intoleranz bei 47 % mit Colchicin vs. 15 % ohne Colchicin
Adler et al. [70]	Europäische ESC-Leitlinie	Akut: 0,5 mg Colchicin täglich (wenn < 70 kg) oder 0,5 mg Colchicin 2‑mal/Tag (wenn ≥ 70 kg) Prophylaxe: 0,5 mg Colchicin täglich (wenn < 70 kg) oder 0,5 mg Colchicin 2‑mal/Tag (wenn ≥ 70 kg)	Akut: 3 Monate, ergänzend zu ASS/NSAID Prophylaxe: ≥ 6 Monate, ergänzend zu ASS/NSAID	First-Line für Akuttherapie und Prophylaxe, ergänzend zu ASS/NSAID	–
O’Gara et al. [89]	Amerikanische ACC/AHA-Leitlinie	Akut: 0,5 mg Colchicin täglich (wenn < 70 kg) oder 0,5 mg Colchicin 2‑mal/Tag (wenn ≥ 70 kg) Prophylaxe: 0,5 mg Colchicin täglich (wenn < 70 kg) oder 0,5 mg Colchicin 2‑mal/Tag (wenn ≥ 70 kg)	Akut: 3 Monate, ergänzend zu ASS/NSAID Prophylaxe: ≥ 6 Monate, ergänzend zu ASS/NSAID	First-Line für Akuttherapie und Prophylaxe, ergänzend zu ASS/NSAID	–

*AHA* American Heart Association, *ASS* Acetylsalicylsäure, *ESC* European Society of Cardiology, *NSAID* nichtsteroidale antiinflammatorische Arzneistoffe, *UAW* unerwünschte Arzneimittelwirkung, *VHF* Vorhofflimmern

### Empfehlungen zur Therapie

Die additive Colchicin-Behandlung richtet sich nicht nach Entzündungsmarkern oder Symptomen, sondern ist bei akuter Perikarditis für 1 bis 3 Monate und bei rezidivierender Perikarditis für mindestens 6 Monate indiziert [70]. Patienten mit akuter Perikarditis und einem Körpergewicht unter 70 kg sollen 1‑mal täglich 0,5 mg Colchicin, Patienten mit einem Körpergewicht ≥ 70 kg 1‑mal täglich 1 mg Colchicin erhalten [70]. Ein Ausschleichen der Dosis am Behandlungsende ist nicht obligat, aber therapeutisch sinnvoll [70], beispielsweise mit 0,5 mg Colchicin jeden zweiten Tag (< 70 kg) oder 1‑mal täglich 0,5 mg Colchicin (≥ 70 kg) in den letzten Wochen [70]. Für therapierefraktäre Patienten, die auch nach Zugabe niedrig dosierter Glukokortikoide nicht ansprechen, ist eine Umstellung auf den selektiven IL-1-Inhibitor Anakinra angezeigt [70]. Dass Colchicin hierzu nicht unbedingt abgesetzt werden muss, illustriert eine multizentrische, retrospektive Beobachtungsstudie bei Glukokortikoid-abhängiger und Colchicin-resistenter rezidivierender Perikarditis [87]. Patienten die zusätzlich zu Anakinra mit Colchicin behandelt wurden, hatten eine geringere Rezidivrate und ein längeres ereignisfreies Überleben [87].

Bei einem Teil der Patienten ist neben dem Herzbeutel auch der Herzmuskel (Myokard) betroffen, wobei das inflammatorische Geschehen entweder vom Herzbeutel auf den Herzmuskel (Myoperikarditis) oder umgekehrt vom Herzmuskel auf den Herzbeutel (Perimyokarditis) übergreift [70]. Beide Krankheitsbilder teilen eine gemeinsame Ätiologie [90, 91]. In der Praxis treten deshalb oft überlappende Formen auf, die sich ohne kardialer Magnetresonanztomographie (MRT) nur schwer voneinander trennen lassen [90, 91]. Vielen Myokarditiden geht eine akute Atemwegserkrankung oder Gastroenteritis voraus [90, 91]. Das klinische Vollbild beinhaltet direkte toxische Auswirkungen auf das Herz und die systemische Aktivierung des Immunsystems [90, 91]. Ein antiinflammatorischer therapeutischer Ansatz erscheint daher naheliegend. Die ESC empfiehlt eine empirische entzündungshemmende NSAR-Therapie in der Erstlinie und Glukokortikoide in der Zweitlinie, sollten NSAR kontraindiziert, unverträglich oder unwirksam sein (Klasse IIa, Evidenzgrad C) [70]. Einige Arbeiten raten bei Myoperikarditis gleichwohl zu einer reduzierten NSAR-Dosis, da sich NSAR in Tiermodellen bei Myokarditis als ineffektiv erwiesen haben und die Mortalität unter Umständen erhöhen [92, 93].

Es gibt aber auch Parallelen zur reinen Perikarditis. So war in der akuten Phase einer Coxsackie-Virus-3-verursachten Myokarditis bei Mäusen das NLRP3-Inflammasom aktiviert [94]. Wurde den Tieren Colchicin verabreicht, verringerte sich die NLRP3-Inflammasomaktivität im Herzen und in der Milz der Mäuse, ohne dass sich die Virusbelastung verschlimmerte [94]. Dies resultierte in einer verbesserten linksventrikulären Funktion [94]. In einer retrospektiven Kohortenstudie mit Patienten, die erstmalig wegen einer idiopathischen/viralen Perikarditis mit myokardialer Beteiligung stationär aufgenommen wurden, hatten jene, die additiv Colchicin erhielten, eine geringere Inzidenz von Rezidiven (19,2 % vs. 43,8 %) und ein längeres ereignisfreies Überleben als Patienten ohne Colchicin [95].

### Dressler-Syndrom

Eine Sonderform der Perikarditis ist die 2 bis 8 Wochen nach einem akuten ACS auftretende Post-MI-Perikarditis (Dressler-Syndrom), deren Inzidenz nach Einführung der primären Revaskularisation deutlich zurückgegangen ist [88]. Wenngleich für diese seltene Erkrankung Daten aus randomisiert kontrollierten Studien fehlen, schließen die ESC (Empfehlung der Klasse IIa, Evidenzgrad B) und das American College of Cardiology (Empfehlung der Klasse IIb, Evidenzgrad C) Colchicin auf Basis der Studien zu Perikarditis in das empfohlene Therapieschema für das Dressler-Syndrom ein [70, 89]. Die Dosierung orientiert sich an jener zur Perikarditistherapie [70, 89].

## Koronare Herzkrankheit

KHK ist ein pathologischer Prozess, der durch die Ansammlung atherosklerotischer Plaques in den epikardialen Arterien gekennzeichnet ist [96]. Die Krankheit kann lange, stabile Phasen haben, allerdings auch jederzeit instabil werden – typischerweise aufgrund eines akuten atherothrombotischen Ereignisses [96]. Diese Ereignisse haben ihren Ursprung in der fortschreitenden Atherosklerose am pathologisch veränderten Gefäßendothel, im Zuge dessen myeloische Zellen lipidreiche Plaques destabilisieren und eine Ruptur oder Erosion verursachen [97]. Kardiovaskuläre Risikofaktoren wie Bluthochdruck, Fettstoffwechselstörung, Diabetes und entzündliche Gelenkerkrankungen (z. B. Gicht) beschleunigen die endotheliale Dysfunktion und setzen einen inflammatorischen Prozess in Gang, der die frühe Phase und Progression der Atherosklerose einleitet [98, 99]. Worst-Case-Szenario ist ein ACS ohne ST-Elevation („non ST-elevation myocardial infarction“ [NSTEMI]) bei unvollständigem Koronarverschluss bzw. mit ST-Elevation („ST-elevation myocardial infarction“ [STEMI]) bei komplettem Koronarverschluss [100, 101]. Überlebende haben ein hohes Risiko für wiederkehrende atherothrombotische Ereignisse [97].

Die vasodilatatorischen, fibrinolytischen und entzündungshemmenden Eigenschaften eines gesunden Koronarendothels schützen vor KHK und ACS [102]. Im erkrankten Zustand werden jedoch die Migration, Adhäsion und Aktivierung neutrophiler Granulozyten und anderer Leukozyten begünstigt [103]. Die von diesen Zellen freigesetzten Enzyme machen die Gefäßwände anfälliger für atherosklerotische Plaques und erzeugen eine unterschwellige Entzündung [104–107]. Blutplättchen wiederum haften sich an die freiliegenden Kollagenfasern an und aggregieren mit zirkulierenden weißen Blutkörperchen, was letztlich die Wahrscheinlichkeit für KHK und ACS erhöht [108]. Seit Bekanntwerden der kausalen Beziehung zwischen Atherosklerose und dem lokalem Entzündungsgeschehen wird hochsensitives CRP (hsCRP) für die Risikobewertung kardiovaskulärer Erkrankungen eingesetzt [109]. Einige in der Sekundärprophylaxe etablierte Medikamente wie Statine senkten in Studien nicht nur das LDL‑C, sondern auch hsCRP, wobei eine stärkere hsCRP-Reduktion mit einer größeren relativen Risikoreduktion für kardiovaskuläre Ereignisse verbunden war [110]. Folgestudien zeigen, dass dieser protektive Effekt unabhängig vom LDL-C-Wert ist [111]. Weil die Bildung und Freisetzung von hsCRP IL‑1 und IL‑6 erfordert, könnte die Blockade dieser Zytokine einen positiven Einfluss auf atherosklerotische Erkrankungen haben [112]. Bestärkt wird diese Annahme durch Arbeiten zum NLRP3-Inflammasom die veranschaulichen, dass Cholesterinkristalle in allen Stadien der Atherosklerose vorhanden sind und diese bei Aktivierung des Immunsystems inflammatorische Schäden induzieren, was das Fortschreiten und die Instabilität von Plaques vorantreibt [113, 114]. Zusammengenommen unterstützen diese Daten den IL-1β/IL-6/CRP-Signalweg als wichtiges therapeutische Ziel zur Bekämpfung kardiovaskulärer Entzündungen [115]. Den endgültigen Beweis lieferte schließlich die CANTOS-Studie, in der die Therapie mit dem selektiven IL-1β-Antagonisten Canakinumab bei Patienten mit früherem ACS und erhöhtem hsCRP-Spiegel die Rate wiederkehrender kardiovaskulärer Ereignisse gegenüber Placebo signifikant verringerte [116]. Dieser Nutzen war unabhängig von der Senkung des Lipidspiegels durch die Komedikation [116]. Wermutstropfen der Canakinumab-Therapie sind die hohen Kosten von rund 11.500 €/Monat (KKP Ö/Stand 9/2024).

Im Unterschied zu Canakinumab wirkt Colchicin (monatlich 43,75 € KKP Ö/Stand 9/2024) über mehrere Mechanismen antiinflammatorisch. Unter anderem wurde festgestellt, dass es die durch oxidativen Stress verursachte Seneszenz und den Seneszenz-assoziierten sekretorischen Phänotyp hemmt, was die Bildung atherosklerotischer Plaques vermindert [117]. Im Endothel verringert Colchicin die Expression von Selektinen, die mit der Adhäsion und dem Eintritt von Leukozyten in Verbindung stehen (vgl. Abb. 2; [118]). Niedrig dosiertes Colchicin (0,5 mg/Tag) reduziert bei Patienten mit KHK den hsCRP-Spiegel um durchschnittlich 40 % und den IL-6-Spiegel um 16 % [119]. Ferner hat es als Add-on zu einer ASS- und Statintherapie positive Auswirkungen auf das Plaquevolumen in Koronargefäßen bei KHK-Patienten nach ACS [120]. Dazu gesellt sich eine stetig wachsende Zahl randomisiert kontrollierter Studien, in denen niedrig dosiertes Colchicin das Risiko ischämischer Ereignisse ungeachtet der Basismedikation verringert [121]. In Tab. 4 sind die Studiendaten von Colchicin bei stabiler KHK dargestellt.

**Tab. 4:** *Studiendaten koronare Herzkrankheit. *Auflistung von Studien die den Einsatz von Colchicin bei koronarer Herzerkrankung untersucht haben unter Berücksichtigung der klinischen Situation, eingesetzten Dosierung, Anwendungsdauer, des Outcomes sowie der häufigsten Nebenwirkung

Studien	Klinische Situation	Dosierung	Anwendungsdauer	Ergebnis	Häufigste UAW unter Colchicin
Nidorf et al. [135]	Stabile KHK mit erhöhtem hsCRP	0,5 mg Colchicin 2‑mal/Tag plus ASS plus Atorvastatin	1 Monat	Reduktion von hsCRP von 4,58 auf 1,78 mg/l	Keine klinisch relevanten
Nidorf et al. [137]	Stabile KHK	0,5 mg Colchicin/Tag plus Statine und Standardmedikation	36 Monate	Reduktion kardiovaskulärer Ereignisse um 16 % mit Colchicin vs. 5,3 % ohne Colchicin	Gastrointestinale Intoleranz bei 2,5 %
Nidorf et al. [134]	Stabile KHK	0,5 mg Colchicin/Tag plus Statine und Standardmedikation	28,6 Monate	Reduktion kardiovaskulärer Ereignisse um 9,6 % mit Colchicin vs. 6,8 % ohne Colchicin	Myalgie bei 21,2 % vs. 18,5 % ohne Colchicin

*hsCRP* Hochsensitives C‑reaktives Protein, *UAW* Unerwünschte Arzneimittelwirkung

### Empfehlungen zur Therapie

Die daraus gewonnenen Daten mündeten in der Zulassung von Colchicin durch die US-amerikanische Arzneimittelbehörde FDA zur Prophylaxe von Myokardinfarkt, Schlaganfall, koronarer Revaskularisation und kardiovaskulärem Tod bei erwachsenen Patienten mit bestehender atherosklerotischer Erkrankung oder mit mehreren Risikofaktoren für Herz-Kreislauf-Erkrankungen [122]. Europäische kardiologische Gesellschaften befürworten diese Vorgehensweise. In den kürzlich von der ESC aktualisierten Leitlinien zum Management des chronischen Koronarsyndroms von 2024 wurde der Empfehlungsgrad für Colchicin von ehemals IIb auf nunmehr IIa („should be considered“) hochgestuft [123]. Demnach sollte bei KHK-Patienten mit atherosklerotischer koronarer Gefäßerkrankung eine niedrig dosierte Colchicin-Therapie (0,5 mg/Tag) in Betracht gezogen werden, um Myokardinfarkt, Schlafanfall und die Notwendigkeit einer Revaskularisierung zu reduzieren [123] – eine Maßnahme, für die auch der nationale italienische Verband der Krankenhauskardiologen (ANMCO) in seinem Positionspapier plädiert [124]. Der Einsatz von Colchicin zur Prävention der KHK und daraus resultierender Krankheitsbilder wird in zahlreichen Übersichtsarbeiten und Konsensuserklärungen thematisiert [125–132].

**Abb. 3:**
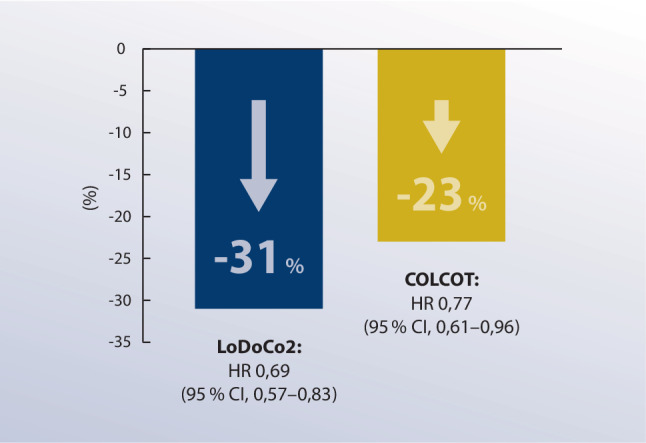
*Hazard Ratios aus LoDoCo2 und COLCOT zur Inzidenz kardiovaskulärer Ereignisse. *LoDoCo2 [134] rekrutierte Patienten mit stabiler koronarer Herzkrankheit unter Basismedikation, COLCOT [140] Patienten mit kürzlich (innerhalb von 30 Tagen) zurückliegendem akutem Koronarsyndrom. Beide Studien randomisierten die Teilnehmer auf Colchicin (0,5 mg/Tag) oder Placebo

Entsprechend der US-Fachinformation stützt sich die Zulassung von Colchicin insbesondere auf die LoDoCo2-Studie mit mehr als 5500 Patienten mit stabiler KHK [134]. Dieser gingen einige hypothesengenerierende Studien mit weniger umfangreichen Patientenzahlen voraus. Nidorf et al. demonstrierten 2007 erstmals, dass niedrig dosiertes Colchicin bei KHK-Patienten unter ASS- und Statintherapie den hsCRP-Spiegel senkt [135]. Crittenden et al. beobachteten eine deutlich geringere ACS-Prävalenz, niedrigere CRP-Werte und eine Tendenz zu einer geringeren Gesamtmortalität bei Gichtpatienten, die Colchicin einnehmen [136]. Mit LoDoCo wurde hierauf die erste klinische Studie zu Colchicin bei Patienten mit KHK realisiert, die als primären Endpunkt die kombinierte Inzidenz von ACS, Herzstillstand außerhalb des Krankenhauses oder nichtkardioembolischem ischämischem Schlaganfall definierte [137]. Dieser wurde durch Colchicin signifikant reduziert (5,3 % vs. 16 %) [137]. Sieben Jahre später folgte die zulassungsrelevante Studie LoDoCo2 [134]. Die randomisierte, doppelblind-placebokontrollierte Studie prüfte, ob 1‑mal täglich 0,5 mg Colchicin den primären Endpunkt – kardiovaskulärer Tod, spontanes ACS, ischämischer Schlaganfall oder Ischämie-bedingte koronare Revaskularisation bei Patienten mit stabiler KHK unter Basismedikation – positiv beeinflusst [134]. Eingeschlossene Patienten (*n* = 8284) waren auf einen Thrombozytenaggregationshemmer oder ein Antikoagulans (99,7 %), einen Lipidsenker (96,9 %), einen Betablocker (62,1 %) und einen Inhibitor des Renin-Angiotensin-Systems (71,7 %) eingestellt [134]. Nach einer medianen Nachbeobachtungszeit von 29 Monaten erreichte Colchicin den primären Endpunkt (HR 0,69 [95 %-CI 0,57–0,83]; *p* < 0,001) mit einer NNT von 36 (vgl. Abb. 3; [134]). Darüber hinaus verminderte es die sekundären Endpunkte schwere unerwünschte kardiovaskuläre Ereignisse, ungeplante koronare Revaskularisierung und individueller Ausgang von ACS [134].

Ein viel diskutierter Kritikpunkt von LoDoCo2 war der Anstieg nichtkardiovaskulärer Todesfälle bei Patienten in der Colchicin-Gruppe, der Anlass zu Sicherheitsbedenken gab [258]. Eine solche Beobachtung machte man weder in COLCOT noch einer anderen kardiovaskulären Endpunktstudie oder Metaanalyse [258]. Eine Folgearbeit beschäftigte sich aus diesem Grund mit den ursachenspezifischen Mortalitätsdaten aus LoDoCo2 [259]. Die Autoren führten eine multivariate Analyse durch und bestimmten Mortalitätsprädiktoren für kardiovaskulären und nichtkardiovaskulären Tod [259]. Von den 133 Todesfällen im Studienzeitraum von LoDoCo2 waren 88 nichtkardiovaskulärer Natur, wovon 53 der Colchicin- und 35 der Placebogruppe angehörten [259]. Diese 88 Todesfälle verteilten sich auf Krebs (26 vs. 22), Lungenerkrankung im Endstadium (9 vs. 4), Infektionen (4 vs. 4), Demenzerkrankungen (4 vs. 1), Multiorganversagen (3 vs. 2) und andere Ursachen (7 vs. 2) [259]. In der multivariablen Analyse war ein Alter über 65 Jahre das einzige unabhängige Ausgangsmerkmal, das mit nichtkardiovaskulärem Tod assoziiert war [259]. Dies unterstreicht die Bedeutung von Komorbiditäten als Treiber der Gesamtmortalität bei Patienten mit chronischer KHK, welche in die Nutzen-Risiko-Abwägung für eine Langzeittherapie mit Colchicin einkalkuliert werden sollten. Daten aus systemischen Reviews von Fallberichten, Arzneimittelregistern, randomisiert kontrollierten Studien und Metaanalysen explizieren, dass die kontinuierliche, langfristige Gabe von niedrig dosiertem Colchicin sicher und abgesehen von leichtem Durchfall zu Therapiebeginn gut verträglich ist [261]. Auswirkungen auf Leber‑, Nieren- und kognitive Funktion, Blutungen, Wundheilung, Fruchtbarkeit, Schwangerschaft, maligne Erkrankungen und schwere Infektionen sind bei ordnungsgemäß verwendetem Colchicin nicht zu erwarten und auf Placeboniveau [261].

## Akutes Koronarsyndrom

Unter Berücksichtigung der myokardialen Ischämien und Plaqueinstabilität zugrunde liegenden Entzündungsreaktion wurde die Gabe von Colchicin auch unmittelbar nach einem ACS untersucht. In der Pilotstudie von Raju et al. mit 82 Patienten nach ACS oder Schlaganfall gelang es Colchicin allerdings nicht, den hsCRP-Spiegel nach 30 Tagen zu senken [138]. Demgegenüber reduzierte Colchicin bei Patienten nach STEMI in einer Initialdosis von 2 mg, gefolgt von 0,5 mg 2‑mal täglich für 5 Tage, die Plasmakonzentration der CK-MB, einem anerkannten diagnostischen Marker eines akuten ACS, und die Infarktgröße gemäß kardialer MRT [139]. Bei Patienten mit kürzlich (< 1 Monat) zurückliegenden ACS hatte eine optimierte Basistherapie plus niedrig dosiertes Colchicin einen remodellierenden Effekt auf die atherosklerotischen Plaques der Herzkranzgefäße in der CT-Koronarangiographie [120]. Trotz vielversprechender Resultate ist die Aussagekraft dieser Pilotstudien aufgrund der geringen Teilnehmerzahl eingeschränkt. Das änderte sich im COLchicine Cardiovascular Outcomes Trial (COLCOT), der 4745 Patienten nach kürzlich zurückliegendem ACS einschloss [140]. Die Teilnehmenden wurden 1:1 auf 0,5 mg Colchicin oder Placebo randomisiert und bis zu 4 Jahre nachbeobachtet [140]. Colchicin verringerte das relative Risiko für kardiovaskuläre Ereignisse signifikant um 23 % (vgl. Abb. 3; [140]). Eine Subanalyse ergab, dass Patienten am meisten profitierten, wenn sie Colchicin innerhalb der ersten 3 Tage nach ACS erhielten [141]. Die vorteilhaften Auswirkungen einer frühen Therapieeinleitung erstreckten sich auf sämtliche Parameter des kombinierten primären Endpunktes (kardiovaskulärer Tod, Herzstillstand, ACS, Schlaganfall, dringender Krankenhausaufenthalt wegen Angina pectoris) [141].

Shah et al. setzten noch einen Schritt davor an und analysierten die entzündungshemmende Wirkung einer akuten Gabe von 1,8 mg Colchicin vs. Placebo auf nachfolgende Myokardschäden vor einer perkutanen Koronarintervention (PCI) [142]. Rund die Hälfte der Probanden, die sich einer PCI unterzogen, taten dies wegen eines ACS [142]. Primärer Endpunkt war das kombinierte Ergebnis aus Tod, nichttödlichem Myokardinfarkt und Revaskularisierung des Zielgefäßes nach 30 Tagen sowie eines PCI-bedingten Myokardinfarkts [142]. Die Studie verlief negativ: Colchicin hatte keinen Einfluss auf die PCI-bedingte Myokardschädigung, schwächte aber den Anstieg der Entzündungsparameter IL‑6 und hsCRP stärker ab als Placebo [142]. Imazio und Nidorf argumentieren in ihrem Review, dass dies womöglich der vergleichsweise späten Colchicin-Gabe nach Entwicklung eines Infarktes sowie der fehlenden Kontrolle für die Komplexität des koronaren Stentings geschuldet ist, was letzten Endes zu einem erhöhten Risiko für Myokardverletzungen durch Atheroembolien geführt haben könnte [9].

Der multizentrische australische COPS-Trial schloss Patienten mit ACS ein, bei denen die Koronarangiographie Hinweise auf eine KHK lieferte [143]. Diese Patienten erhielten Colchicin (0,5 mg 2‑mal täglich im ersten Monat, gefolgt von 0,5 mg täglich für 11 Monate) oder Placebo ergänzend zur standardmäßigen medikamentösen Sekundärprävention oder PCI und wurden für mindestens 12 Monate nachbeobachtet [143]. In diesem Zeitraum gab es 24 Ereignisse in der Colchicin-Gruppe verglichen zu 38 Ereignissen in der Placebogruppe (nicht signifikant) [143]. Im 2‑Jahres-Follow-up war Colchicin Placebo mit 32 vs. 54 Ereignissen überlegen (signifikant) [144]. Unerwünschte Begleiterscheinungen einschließlich Nebenwirkungen gastrointestinaler Natur unterschieden sich in beiden Gruppen nicht voneinander [144, 145].

Die prospektive doppelblinde Studie von Akrami et al. rekrutierte 249 Patienten mit koronarangiographisch bestätigten akuten ACS und randomisierte diese auf Colchicin oder Placebo [146]. Ziel war es herauszufinden, ob Colchicin den primären Endpunkt MACE (ACS, dekompensierte Herzinsuffizienz, Schlaganfall) additiv zur medikamentösen Standardtherapie reduziert [146]. Über einen Zeitraum von 6 Monaten traten 36 schwere kardiovaskuläre Ereignisse auf, davon 8 in der Colchicin-Gruppe und 28 in der Placebogruppe [146]. COLD-MI beurteilte den Effekt von Colchicin (1 mg innerhalb der ersten 48 h) auf die Myokarddenervierung bei reperfundierten ACS-Patienten [147]. Dazu wurde der Verlust der präsynaptischen Adrenalin-Wiederaufnahmefunktion mithilfe der 123I-Metaiodobenzylguanidin-Einzelphotonen-Emissions-CT beurteilt [147]. Colchicin illustrierte dabei eine starke Schutzwirkung auf die Myokarddenervierung in Zonen ohne Infarkt (Odds Ratio [OR] 0,43 [95 %-KI 0,19–0,96]) als auch in Zonen mit Infarkt (OR 0,54 [95 %-KI 0,14–1,20]) [147]. In der Pilotstudie MACT wurde Colchicin mit Ticagrelor oder Prasugrel bei Patienten nach PCI kombiniert und die Stentthromboserate nach 3 Monaten bewertet [148]. Dieses Vorgehen war mit einer besseren Thrombozytenfunktion und einem günstigeren Entzündungsprofil als Standard-ASS assoziiert [148]. Eine weitere Pilotstudie verglich niedrig dosiertes Colchicin (0,5 mg 1‑mal täglich) mit 2 anderen entzündungshemmenden Arzneistoffen (Tranilast: 0,1 g 3‑mal täglich; Oridonin: 0,5 g 3‑mal täglich) bei Patienten nach PCI [149]. Der primäre Endpunkt einer vordefinierten hsCRP-Senkung wurde nur in der Colchicin-Gruppe erreicht [149]. Eine gezielte Proteomanalyse ergab, dass Colchicin Proteine, die mit der Neutrophilenaktivierung, der Blutplättchenaggregation und der Endothelschädigung zusammenhängen, positiv moduliert [149].

Diesen optimistischen Resultaten steht der negative Ausgang von COVERT-MI gegenüber, wo die orale Gabe von hoch dosiertem Colchicin zum Zeitpunkt der Reperfusion und über 5 Tage nach akutem STEMI die Infarktgröße in der kardialen MRT im Vergleich zu Placebo nicht verringerte und sogar mit einer Zunahme linksventrikulärer Thromben verbunden war [150]. Auch im 1‑Jahres-Follow-up gab es keinen signifikanten Unterschied zwischen Colchicin und Placebo [151]. Trotz des anfänglichen Anstiegs linksventrikulärer Thromben in der Colchicin-Gruppe wurden keine übermäßigen ischämischen Nebenwirkungen beobachtet [151].

Zusammengefasst sind die bisherigen Daten für eine breite Colchicin-Anwendung beim akuten Koronarsyndrom inkongruent (vgl. Tab. 5). Therapieempfehlungen von Fachgesellschaften stehen infolgedessen aus, wenngleich die entzündungshemmende Komponente nicht außer Acht gelassen werden darf, wie in CANTOS eindrücklich dargestellt [116]. Eventuell ändert sich das durch CLEAR SYNERGY (NCT03048825). Die Studie untersucht die Langzeittherapie von Colchicin (allein oder in Kombination mit Spironolacton) bei ACS-Patienten nach PCI hinsichtlich schwerer kardiovaskulärer Ereignisse, kardiovaskulär bedingtem Tod und Herzinsuffizienz. Erste Ergebnisse werden 2025 erwartet.

**Tab. 5:** *Studiendaten akutes Koronarsyndrom. *Auflistung von Studien, die den Einsatz von Colchicin beim akuten Koronarsyndrom untersucht haben unter Berücksichtigung der klinischen Situation, eingesetzten Dosierung, Anwendungsdauer, des Outcomes sowie der häufigsten Nebenwirkung

Studien	Klinische Situation	Dosierung	Anwendungsdauer	Ergebnis	Häufigste UAW unter Colchicin
Raju et al. [138]	ACS oder Schlaganfall	1,0 mg Colchicin/Tag	1 Monat	Kein Unterschied zwischen Colchicin und Placebo hinsichtlich hsCRP-Reduktion	Durchfall bei 14 Patienten in der Colchicin- vs. 7 Patienten in der Placebogruppe
Deftereos et al. [139]	STEMI	2,0 mg Colchicin am ersten Tag gefolgt von 0,5 mg Colchicin 2‑mal/Tag	5 Tage	Reduktion der CK-MB um 3,14 ng/ml mit Colchicin vs. 6,18 ng/ml mit Placebo Reduktion der Infarktgröße gemäß kardialer MRT um 18,3 ml/1,73 m^2^ mit Colchicin vs. 23,2 ml/1,73 m^2^ mit Placebo	Durchfall bei 15 Patienten in der Colchicin- vs. 1 Patienten in der Placebogruppe
Vaidya et al. [120]	ACS	0,5 mg Colchicin/Tag plus optimierte Standardtherapie	12 Monate	Reduktion des kardialen Plaquevolumens um 15,9 mm^3^ mit Colchicin vs. 6,6 mm^3^ mit Placebo Reduktion des hsCRP um 1,10 mg/l mit Colchicin vs. 0,38 mg/l mit Placebo	Durchfall bei 1 Patienten in der Colchicin- vs. 0 Patienten in der Placebogruppe
Tardif et al. [140]	ACS	0,5 mg Colchicin/Tag plus Standardtherapie	20 Monate	Reduktion kardiovaskulärer Ereignisse um 7,1 % mit Colchicin vs. 5,5 % mit Placebo	Durchfall bei 17,5 % vs. 17,6 % mit Placebo
Shah et al. [142]	PCI (davon 50 % aufgrund ACS)	1,8 mg Colchicin prä-interventionell	Einmalig	Kein Unterschied hinsichtlich PCI-bedingter Myokardschädigung zu Placebo (57,3 % vs. 64,2 %)	Gastrointestinale Intoleranz 9,3 % vs. 3,2 % mit Placebo
Tong et al. [143, 144]	ACS	0,5 mg Colchicin 2‑mal/Tag im ersten Monat gefolgt von 0,5 mg Colchicin/Tag	12 Monate	Nicht-signifikante Reduktion kardiovaskulärer Ereignisse nach 12 Monaten (24 vs. 38) Signifikante Reduktion kardiovaskulärer Ereignisse nach 24 Monaten (32 vs. 54)	Gastrointestinale Intoleranz 23,0 % vs. 20,8 % mit Placebo
Akrami et al. [146]	ACS	0,5 mg Colchicin/Tag plus Standardtherapie	6 Monate	Weniger kardiovaskuläre Ereignisse mit Colchicin vs. Placebo (8 vs. 28)	Durchfall bei 15 Patienten in der Colchicin- vs. 3 Patienten in der Placebogruppe
Huet et al. [147]	ACS	1,0 mg Colchicin innerhalb von 48 h	1 Monat	Schutzwirkung von Colchicin auf die Myokarddenervierung in Zonen mit und ohne Infarkt vs. kein Colchicin	Gastrointestinale Intoleranz 51,8 % vs. 18,5 % ohne Colchicin
Lee et al. [148]	ACS + DES	0,6 mg Colchicin einen Tag nach PCI plus Ticagrelor oder Prasugrel	3 Monate	Bessere Thrombozytenfunktion und günstigeres Entzündungsprofil mit Colchicin vs. ASS	Nicht erhoben
Yu et al. [149]	PCI ± ACS	0,5 mg Colchicin/Tag	1 Monat	Reduktion des hsCRP um 48,28 % mit Colchicin vs. 21,6 % mit Tranilast vs. 11,62 % mit Placebo vs. 7,81 % Oridonin	Nicht erhoben
Mewton et al. [150]	STEMI + PCI	2,0 mg Colchicin kurz nach PCI gefolgt von 0,5 mg Colchicin 2‑mal/Tag	5 Tage	Kein Unterschied hinsichtlich Reduktion der Infarktgröße im kardialen MRT zwischen Colchicin und Placebo	Gastrointestinale Intoleranz 34,4 % vs. 10,1 % mit Placebo

*ACS* akutes Koronarsyndrom, *CK-MB* Kreatinkinase Muscle-Brain-Type, *DES* Drug-eluting-Stent, *hsCRP* hochsensitives C‑reaktives Protein, *PCI* perkutane Koronarintervention, *STEMI* ST-Elevationsinfarkt, *UAW* unerwünschte Arzneimittelwirkung

## Vorhofflimmern

Mit einer Prävalenz von ungefähr 3 % zählt Vorhofflimmern (VHF) zu den häufigsten Herzrhythmusstörungen [152]. Man schätzt, dass jeder dritte heute 55-jährige Mensch im Laufe seines Lebens erkranken wird [152]. VHF geht mit einer beträchtlichen Belastung für die Patienten und das Gesundheitssystem einher [152]. Die abnormale, schnelle und unregelmäßige Kontraktion der Herzmuskelzellen verursacht Symptome wie Palpitationen und Schwindel – im schlimmsten Fall ist sie Auslöser schwerer kardiovaskulärer Erkrankungen wie Herzinsuffizienz und Schlaganfall [153]. Zu den Risikofaktoren gehören Bluthochdruck, KHK, Herzklappenerkrankungen, Alkoholkonsum, Übergewicht und Hyperthyreose [153]. Wachsende Evidenz spricht dafür, dass auch Entzündungen und damit assoziierte kardiale Umbauvorgänge als Hauptakteure fungieren [154, 155]. In Beobachtungsstudien standen erhöhte CRP-Spiegel mit persistierendem VHF, positiver VHF-Anamnese und der Entwicklung von VHF nach myokardialer Revaskularisation mit/ohne koronararterielle/r Bypassoperation (CABG) in Zusammenhang [156–158]. Proinflammatorische Zytokine und oxidativer Stress fördern den elektrischen und strukturellen Umbau der Vorhöfe und damit Vorhofektopie, Vorhofarrhythmien und interstitielle Fibrose [159].

Yao et al. demonstrierten anhand eines Immunoblots in atrialen Gewebelysaten und Kardiomyozyten von Patienten mit paroxysmalen oder chronischen VHF eine Aktivierung des NLRP3-Inflammasoms [160]. In ihrem Kardiomyozyten-spezifischen Knock-In-Mausmodell mit überexprimierten NLRP3 entwickelten Mäuse spontane vorzeitige Vorhofkontraktionen und induzierbares VHF, das durch einen spezifischen NLRP3-Inflammasom-Inhibitor abgeschwächt wurde [160]. Unbehandelte Mäuse hatten hingegen eine abnormale Kalziumfreisetzung aus dem sarkoplasmatischen Retikulum, eine verkürzte effektive Refraktärzeit und atriale Hypertrophie [160]. Die genetische Hemmung von NLRP3 verhinderte das Auftreten von VHF in transgenen CREM-Mäusen, einem gut charakterisierten Mausmodell für spontanes Vorhofflimmern [160].

Colchicin unterdrückt die beschriebenen Entzündungsvorgänge, indem es die Migration, Adhäsion und Aktivierung von Neutrophilen, die Freisetzung von Defensinen, Matrixmetalloproteinasen und Elastin durch Neutrophile, die Aktivierung des Entzündungswegs durch NLRP3 sowie die Proliferation der glatten Muskulatur und Gefäßstenose hemmt [161]. Dies macht Colchicin zu einem attraktiven Arzneistoff für die Behandlung von VHF. Die Tab. 6 fasst die nachfolgend beschriebenen Studien zum Einsatz von Colchicin bei VHF zusammen.

**Tab. 6:** *Studiendaten Vorhofflimmern. *Auflistung von Studien, die den Einsatz von Colchicin bei Vorhofflimmern untersucht haben unter Berücksichtigung der klinischen Situation, eingesetzten Dosierung, Anwendungsdauer, des Outcomes sowie der häufigsten Nebenwirkung

Studien	Klinische Situation	Dosierung	Anwendungsdauer	Ergebnis	Häufigste UAW unter Colchicin
Imazio et al. [166]	VHF nach Herz-OP	1,0 mg Colchicin am dritten postoperativen Tag gefolgt von 0,5 mg Colchicin/Tag (wenn < 70 kg) oder 0,5 mg Colchicin 2‑mal/Tag (wenn ≥ 70 kg)	1 Monat	Reduktion von POAF um 22 % mit Colchicin vs. 12 % ohne Colchicin	Gastrointestinale Intoleranz bei 9,5 % vs. 4,2 % ohne Colchicin
Imazio et al. [167]	VHF nach Herz-OP als sekundärer Endpunkt	0,5 mg Colchicin/Tag (wenn < 70 kg) oder 0,5 mg Colchicin 2‑mal/Tag (wenn ≥ 70 kg) beginnend 48–72 h vor der Operation	1 Monat	Auftreten von POAF bei 41,7 % der Patienten ohne Colchicin vs. 33,9 % der Patienten mit Colchicin	Gastrointestinale Intoleranz bei 14,4 % vs. 6,7 % ohne Colchicin
Tabbalat et al. [168]	VHF nach Herz-OP	2,0 mg Colchicin 12–24 h vor der Operation gefolgt von 0,5 mg Colchicin 2‑mal/Tag (oder halbe Dosis, wenn < 70 kg) bis zur Entlassung aus dem Krankenhaus	8,3 Tage	Auftreten von POAF bei 20,5 % der Patienten ohne Colchicin vs. 14,5 % der Patienten mit Colchicin	Durchfall bei 24,6 % vs. 5,5 % ohne Colchicin
Zarpelon et al. [169]	VHF nach Herz-OP	1,0 mg Colchicin 2‑mal/Tag vor der Operation gefolgt von 0,5 mg Colchicin 2‑mal/Tag bis zur Entlassung aus dem Krankenhaus	2 Wochen	Auftreten von POAF bei 13 % der Patienten ohne Colchicin vs. 7 % der Patienten mit Colchicin	Infektionsrate bei 26,8 % vs. 11,8 % ohne Colchicin
Shvartz et al. [170]	VHF nach Herz-OP	1,0 mg Colchicin/Tag beginnend am Tag vor der Operation bis zum fünften postoperativen Tag	5 Tage	Auftreten von POAF bei 30,7 % der Patienten ohne Colchicin vs. 18,6 % der Patienten mit Colchicin	Durchfall bei 25,7 % vs. 11,8 % ohne Colchicin
Deftereos et al. [172]	VHF-Ablation	0,5 mg Colchicin 2‑mal/Tag beginnend am Tag der Ablation	3 Monate	Auftreten von POAF bei 33,5 % der Patienten ohne Colchicin vs. 16,0 % der Patienten mit Colchicin CRP an Tag 4 0,46 mg/l mit Colchicin vs. 1,18 mg/l ohne Colchicin IL‑6 an Tag 4 0,10 pg/ml mit Colchicin vs. 0,50 pg/ml ohne Colchicin	Durchfall bei 8,6 % vs. 1,3 % ohne Colchicin
Deftereos et al. [173]	VHF-Ablation	0,5 mg Colchicin 2‑mal/Tag beginnend am Tag der Ablation	3 Monate	Auftreten von POAF bei 49,5 % der Patienten ohne Colchicin vs. 31,1 % der Patienten mit Colchicin Verbesserte Scores der physischen Domäne der Lebensqualität nach 12 Monaten unter Colchicin	Durchfall bei 9,7 % vs. 1,9 % ohne Colchicin
Egami et al. [174]	VHF-Ablation	0,5 mg Colchicin/Tag nach der Ablation	2 Wochen	Reduktion der Inzidenz von VHF-Rezidiven um 34,2 % mit Colchicin vs. 10,5 % ohne Colchicin bei Patienten mit größerem epikardialem Fettgewebe gemäß MRT	Nicht erhoben
Benz et al. [176]	VHF-Ablation	0,6 mg Colchicin 2‑mal/Tag beginnend innerhalb von 4 h vor der Ablation	10 Tage	Inzidenz für VHF-Rezidive bei 31 % unter Colchicin vs. 32 % unter Placebo	Durchfall bei 26,26 % vs. 7,07 % mit Placebo
Bessissow et al. [177]	VHF nach NCS	0,6 mg Colchicin einige Stunden vor der Operation gefolgt von 0,6 mg Colchicin 2‑mal/Tag	10 Tage	Auftreten von POAF bei 10,2 % mit Colchicin vs. 13,7 % mit Placebo	Durchfall bei 10,2 % vs. 2,0 % mit Placebo
Conen et al. [178]	VHF nach NCS	0,5 mg Colchicin 2‑mal/Tag beginnend innerhalb von 4 h vor der Operation	10 Tage	Auftreten von POAF bei 18,3 % mit Colchicin vs. 20,3 % mit Placebo	Durchfall bei 8,3 % vs. 2,4 % mit Placebo

*NCS* nichtkardial-chirurgischer Eingriff, *POAF* postoperatives Vorhofflimmern, *UAW* unerwünschte Arzneimittelwirkung, *VHF* Vorhofflimmern

### Postoperatives Vorhofflimmern nach Herzoperation

Neu aufgetretenes postoperatives VHF (POAF) ist die häufigste Komplikation nach Herzoperationen und verantwortlich für längere und wiederholte Krankenhausaufenthalte nach dem Eingriff [162]. Die Inzidenz für Patienten nach CABG beträgt 30 %, nach isolierter Klappenoperation 40 % und bei kombiniert operierten Patienten 50 % [163–165]. Erste Resultate zum Einsatz von Colchicin gegen POAF stammen aus einer Substudie (COPPS-POAF) des COlchicine for the Prevention of the Post-pericardiotomy Syndrome (COPPS) Trials, in der Colchicin die Inzidenz von POAF nach 30 Tagen gegenüber Placebo verringerte (12 % vs. 22 %) [166]. Hierauf schloss sich COPPS‑2 mit einer ähnlichen Zielsetzung an [167]. Randomisierte Patienten erhielten 48–72 h vor der Operation Colchicin (1 mg 2‑mal täglich, danach 0,5–1 mg/Tag für einen Monat) oder Placebo [167]. Auch hier reduzierte Colchicin die POAF-Inzidenz (33,9 % vs. 41,7 %) [167]. Des Weiteren profitierte die Colchicin-Gruppe von einem niedrigeren CRP- und IL-6-Spiegel [167]. Zwei jüngere Studien mit einem kürzeren Beobachtungszeitraum bei Patienten nach offener Herzoperation oder myokardialer Revaskularisierung verliefen neutral, obwohl man auch hier einen positiven Trend in Richtung Colchicin registrierte [168, 169]. Demgegenüber stehen die Ergebnisse der COCS-Studie, in der Patienten mit Koronararterien-Bypass-Transplantation und/oder Aortenklappenersatz 1‑mal täglich 1 mg Colchicin oder Placebo am Tag vor der Operation sowie an den postoperativen Tagen 2, 3, 4 und 5 erhielten [170]. Die endgültige Analyse umfasst 240 Teilnehmer, davon 113 in der Colchicin- und 127 in der Placebogruppe [170]. POAF wurde bei 21 (18,6 %) Patienten der Colchicin-Gruppe im Vergleich zu 39 (30,7 %) Kontrollpatienten beobachtet [170]. Bei Parametern, die den Schweregrad der Entzündung widerspiegeln (Perikarderguss, Pleuraerguss, Leukozytenzahl, Neutrophilenzahl), schnitt Colchicin in der frühen postoperativen Phase (Tage 3 und 5) ebenfalls besser ab [170]. Durchfall und abdominelle Schmerzen waren unter Colchicin häufiger als unter Placebo [170]. Eine kürzlich publizierte Metaanalyse randomisiert kontrollierter Studien schlussfolgert, dass Colchicin die Inzidenz von POAF bei Patienten nach einer Herzoperation vermindert [171].

### Postablatives Vorhofflimmern

Durch die VHF-Ablationstherapie induzierte proinflammatorische Prozesse stehen mit dem Wiederauftreten von Arrhythmien nach der Ablation in Verbindung. Ob Colchicin in diesem Setting Placebo überlegen ist, testeten 2 randomisiert kontrollierte Studien von Deftereos et al. [172, 173]. In der ersten erhielten Patienten mit paroxysmalen VHF, die sich einer Radiofrequenzablationsbehandlung (RF-Ablation) unterzogen, eine 3‑monatige Behandlung mit 0,5 mg Colchicin 2‑mal täglich oder Placebo [172]. Die Konzentrationen von C‑reaktivem Protein (CRP) und Interleukin(IL)-6 wurden am ersten und vierten Tag der Behandlung gemessen [172]. Im 3‑monatigen Follow-up wurde bei 27 (33,5 %) der 80 Patienten der Placebogruppe ein Wiederauftreten des VHF beobachtet im Vergleich zu 13 (16 %) der 81 Patienten, die Colchicin erhielten [172]. Colchicin reduzierte IL‑6 und CRP stärker als Placebo [172]. In der korrespondierenden zweiten Studie wurden die Patienten im Mittel 15 Monate nachbeobachtet, und ergänzend wurde die Lebensqualität nach 3 und 12 Monaten mittels 26-item World Health Organization QOL-Fragebogen erhoben [173]. Colchicin verringerte die Rezidivrate (31,1 % vs. 49,5 %) und besserte die Scores der physischen Domäne der Lebensqualität [173]. In einer lediglich als Abstract veröffentlichten Studie mit 122 VHF-Patienten, die sich einer Ablation unterzogen, wurde Colchicin in einer Dosierung von 0,5 mg/Tag für 2 Wochen nach der Ablation verordnet und die Häufigkeit von VHF-Rezidiven eruiert [174]. Patienten mit größerem epikardialem Fettgewebe gemäß Multidetektor-CT hatten 12 Monate nach der Ablation eine geringere Inzidenz von VHF (10,5 % vs. 34,2 %), während sich diese bei Patienten mit weniger epikardialem Fettgewebe nicht von Placebo unterschied [174]. Da epikardiales Fettgewebe über entzündungsfördernde Eigenschaften verfügt, wäre das bessere Ansprechen auf Colchicin bei verstärkter kardialer Adipositas eine mögliche Erklärung für die beobachtete Differenz zwischen den beiden Gruppen [175]. Bei Benz et al. konnte Colchicin, das für 10 Tage nach einer Katheterablation verabreicht wurde, das Wiederauftreten von VHF und VHF-assoziierten Ereignissen nicht verhindern [176]. Mohanty et al. teilten 1075 VHF-Patienten, denen ihre erste Katheterablation bevorstand, einer von 3 Gruppen zu [262]. Gruppe 1 erhielt kein Colchicin, Gruppe 2 erhielt Colchicin (0,3 mg 2‑mal täglich) 7 Tage vor bis 1 Monat nach der Ablation, und Gruppe 3 erhielt Colchicin (0,3 mg 2‑mal täglich) am Tag des Eingriffes bis 1 Monat danach [262]. Ziel der Studie war es, die Wirksamkeit von Colchicin in Bezug auf die Vorbeugung einer Perikarditis sowie einer Perikarditis-assoziierten Hospitalisierung zu bewerten [262]. Colchicin reduzierte das Risiko für akute Perikarditiden und damit verbundener Krankenhauseinweisungen deutlich [262]. In der 1‑jährigen Nachbeobachtung stellte sich heraus, dass Colchicin die Überlebensrate verlängerte [262]. Die multizentrische PAPERS-Studie hatte eine vergleichbare Zielsetzung [263]. VHF-Patienten mit bevorstehender RF-Ablation wurden unmittelbar nach dem Eingriff für 7 Tage auf 0,6 mg Colchicin 2‑mal/Tag (*n* = 73) oder Standard-of-Care (*n* = 66) randomisiert mit dem Ziel, die Häufigkeit und Schwere von Perikarditiden innerhalb der ersten 2 Wochen zu verringern [263]. Den primären Endpunkt einer klinischen Perikarditis erreichten 9,6 % in der Colchicin- und 10,6 % in der Standard-of-Care-Gruppe [263]. Magen-Darm-Beschwerden traten unter Colchicin häufiger auf [263].

### Postoperatives Vorhofflimmern nach nichtkardial-chirurgischen Eingriffen

Ob Colchicin die POAF-Inzidenz auch außerhalb der Herzchirurgie vermindert, wollten COP-AF und dessen Vorstudie herausfinden [177, 178]. In die Vorstudie wurden Patienten, die sich einer Lungenresektion unterzogen, eingeschlossen und per Zufall 0,6 mg Colchicin 2‑mal täglich oder Placebo, beginnend einige Stunden vor der Operation, zugeteilt [177]. Die Therapie wurde für weitere 9 Tage beibehalten [177]. Primärer Endpunkt war POAF innerhalb von 30 Tagen nach der Randomisierung [177]. Dieses trat bei 5 (10,2 %) Patienten in der Colchicin-Gruppe und 7 (13,7 %) Patienten in der Placebogruppe auf [177]. Die große internationale COP-AF-Studie schloss daraufhin 3209 Patienten ein, die sich einer größeren nichtkardialen Thoraxoperation unterzogen, und teilte sie 1:1 nach dem Zufallsprinzip einer oralen Gabe von 0,5 mg Colchicin 2‑mal täglich (*n* = 1608) oder Placebo (*n* = 1601) zu [178]. Die Einnahme begann 4 h vor der Operation und wurde über 10 Tage fortgesetzt [178]. Der koprimäre Endpunkt bestand aus POAF und Myokardverletzung in der 14-tägigen Nachbeobachtungszeit [178]. POAF trat bei 6,4 % der mit Colchicin und 7,5 % der mit Placebo behandelten Patienten auf (nicht signifikant) [178]. Bei den Myokardverletzungen verhielt es sich ähnlich (18,3 % vs. 20,3 %) [178].

## Herzinsuffizienz

Herzinsuffizienz (HI) ist gekennzeichnet durch eine myokardiale Dysfunktion variabler Ursache, die sich in einer systolischen, diastolischen oder systolisch-diastolischen Funktionsstörung äußert [179]. Vom Patienten wahrgenommene Beschwerden sind Kurzatmigkeit, eingeschränkte physische Belastbarkeit, Müdigkeit und periphere Ödeme [179]. Die funktionelle Einteilung erfolgt je nach Schwere der Symptome in eine von 4 Klassen der New York Heart Association (NYHA I–IV) [179]. In der westlichen Welt ist ACS die häufigste Ursache für HI [180]. Allein in Österreich erleiden pro Jahr 1,7 % ein ACS oder haben chronische Beschwerden infolge eines ACS [181]. Der regionale Untergang von Herzmuskelgewebe setzt eine Negativspirale mit vielen akuten und chronischen Anpassungen wie neurohumorale Hyperaktivität, kardialer Umbau und Entzündungsreaktion in Gang, die sich schlussendlich zu einer manifesten chronischen HI ausweiten können [182]. Inflammatorische Signalwege sind an der unerwünschten ventrikulären Remodellierung beteiligt, und es ist bekannt, dass entzündliche Zytokine im Blut zirkulieren, welche die klinischen Ergebnisse bei chronischer HI vorhersagen können [183].

In der ACCLAIM-Studie wurde erstmals ein auf die endogene Entzündung abzielender Ansatz an über 2400 Patienten mit NYHA II–IV geprüft [184]. Teilnehmer erhielten eine unspezifische Immunmodulationstherapie mit Eigenblut und wurden für durchschnittlich 10,2 Monate nachbeobachtet [184]. Obwohl es in der gesamten Kohorte keinen Unterschied beim primären Endpunkt Gesamtmortalität oder kardiovaskuläre Aufnahme gab, gelang es in der Subgruppe bei Patienten mit nichtischämischer Kardiomyopathie und milderen NYHA-II-Symptomen, die Gesamtmortalität um 39 % und die Zahl kardiovaskulär bedingter Krankenhausaufnahmen um 26 % zu reduzieren [184]. Dies deutet, zumindest in einer Subpopulation von Patienten, darauf hin, dass die Hemmung der niederschwelligen kardialen Entzündung ein potenziell wirksamer Behandlungsansatz bei chronischer HI ist.

Dieser Ansatz führte zur bislang einzigen randomisiert kontrollierten Studie mit Colchicin bei HI, welche die Wirksamkeit einer 6‑monatigen entzündungshemmenden Colchicin-Behandlung bei Patienten mit stabiler chronischer HI untersuchte [185]. Diese wurden dafür 1:1 auf 0,5 mg Colchicin 2‑mal täglich oder Placebo randomisiert [185]. Primärer Endpunkt war eine Verbesserung um mindestens eine Stufe im NYHA-Funktionsstatus [185]. Das erreichten 14 % der Patienten unter Colchicin und 11 % unter Placebo [185]. Der kombinierte sekundäre Endpunkt aus Tod oder Krankenhausaufenthalt war in beiden Gruppen vergleichbar (9,4 % vs. 10,1 %). Die Serumkonzentrationen von CRP und IL‑6 wurden durch Colchicin, jedoch nicht durch Placebo signifikant verringert [185]. In den 1‑Jahres-Follow-up-Daten der COVERT-MI-Studie, die hoch dosiertes Colchicin zum Zeitpunkt der Reperfusion plus weitere 5 Tage nach STEMI prüfte, gab es einen Trend zu weniger Herzinsuffizienzereignissen in der Colchicin-Gruppe (11,9 % vs. 19,5 % in der Placebogruppe) [151]. Das letzte Wort für Colchicin bei HI ist demnach noch nicht gesprochen (vgl. CLEAR SYNERGY; NCT03048825).

## Schlaganfall

Ein Schlaganfall ist ein akut auftretender medizinischer Notfall, der sich durch fokal neurologische Defizite bemerkbar macht [186]. Auslöser ist eine Schädigung von Gehirngewebe entweder durch einen Gefäßverschluss (ischämischer Schlaganfall) oder eine Blutung (hämorrhagischer Schlaganfall) [186]. Aus epidemiologischen Studien gewonnene Daten veranschaulichen, dass die große Mehrzahl aller Schlaganfälle ischämischer Natur ist [187]. Grund sind meist atherosklerotische kardiovaskuläre Erkrankungen [187]. Pharmakologische Interventionen zur Sekundärprophylaxe fokussieren auf die Hemmung der Thrombozytenaggregation [188]. Obschon dieses Vorgehen zweifelsfrei effektiv ist, erleidet immer noch ein Drittel aller Patienten unter bestehender plättchenhemmender Therapie einen neuerlichen Schlaganfall [189]. Andersartige Therapieansätze sind daher dringend erforderlich, etwa solche, die auf die inflammatorische Komponente wirken. Positronenemissionstomographie(PET-)Studien belegen einen Zusammenhang zwischen Plaqueentzündung, Plaqueruptur und erhöhtem Schlaganfallrisiko [190]. Dem NLRP3-Inflammasom, dessen Expressionsrate bei Stressreizen wie Cholesterinkristallen steigt, kommt große Bedeutung bei der Entstehung und Unterhaltung atherosklerotischer Prozesse zu [113, 191]. NLRP3 bildet Caspase‑1, das seinerseits die atherogenen Zytokine und kardiovaskulären Marker IL-1β und IL-18 hochreguliert [113]. Colchicin verhindert die Aktivierung des NLRP3-Inflammasoms über mehrere zelluläre Mechanismen in Neutrophilen und Makrophagen [192]. Ergo könnte Colchicin die therapeutische Lücke in der Schlaganfallprävention füllen.

Daten aus COLCOT nach einem kürzlichen ACS zeigen ein deutlich geringeres Schlaganfallrisiko in der Colchicin- verglichen mit der Placebogruppe (HR 0,26) [140]. In einer komparativen Studie zur Intima-Media-Dicke-Messung mit insgesamt 102 chronisch kranken Patienten unter Statintherapie und bei Bedarf weiteren Standardmedikamenten wurden 2 Gruppen gebildet [193]: eine Gruppe mit Patienten mit kardiovaskulären Risikofaktoren und Gicht, die Colchicin in einer Dosierung von 0,5 mg 2‑mal täglich einnahmen [193], die zweite Gruppe ohne Gicht und Colchicin [193]. Patienten in Gruppe 1 hatten eine signifikant geringere Intima-Media-Dicke und ein niedrigeres CRP als Patienten in Gruppe 2 [193]. In der kleinen Pilotstudie von Yuan et al. erhielten Patienten mit akutem ischämischem Schlaganfall oder transitorisch ischämischer Attacke (TIA) innerhalb von 24 h nach Symptombeginn Colchicin in einer von 4 Dosen [194]. Die frühzeitige Behandlung mit Colchicin reduzierte signifikant den hsCRP-Spiegel der Patienten [194]. Mehrere Metaanalysen randomisiert kontrollierter Studien an weit über 10.000 Patienten mit atherosklerotisch kardiovaskulärer Erkrankung konstatieren, dass Colchicin die Schlaganfallrate deutlich reduziert, ohne einen Anstieg gastrointestinaler oder muskulärer Nebenwirkungen zu provozieren [195–199]. Die Masse der Patienten stammt aus den Studien COLCOT, LoDoCo, LoDoCo2 und COPS, wo die Schlaganfallrate als einer der primären oder sekundären Endpunkte zu finden ist.

CONVINCE (Colchicine for Prevention of Vascular Inflammation in Noncardioembolic Stroke) bewertete die Anwendung von Colchicin bei mehr als 3000 Erwachsenen über 40 Jahren mit kürzlich zurückliegendem ischämischem Schlaganfall oder TIA, die nicht auf einer Herzembolie oder anderen definierten Ursachen beruhten [200]. Die Ergebnisse wurden im Juni 2024 veröffentlicht. Die Patienten wurden randomisiert einer Behandlung mit 0,5 mg Colchicin pro Tag plus üblicher Pflege (definiert als Thrombozytenaggregationshemmung, lipidsenkende und blutdrucksenkende Behandlung sowie angemessene Ratschläge zum Lebensstil) oder einer alleinigen üblichen Pflege zugeteilt. Der primäre Endpunkt war die Zeit bis zum ersten wiederkehrenden ischämischen Schlaganfall, Myokardinfarkt, Herzstillstand oder Krankenhausaufenthalt mit instabiler Angina pectoris (tödlich oder nicht tödlich). Obwohl die ITT-Analyse den vorgegebenen Schwellenwert für statistische Signifikanz nicht erreichte, verzeichnete die Colchicin-Gruppe zahlenmäßig weniger wiederkehrende Schlaganfälle und koronare Ereignisse. Dieser positive Trend war auch bei sämtlichen sekundären Endpunkten ersichtlich. Patienten unter Colchicin hatten zu allen Messzeitpunkten (28 Tage, 1, 2 und 3 Jahre) einen niedrigeren CRP-Wert (*p* < 0,05 für alle Zeitpunkte). Nach Meinung der Autoren unterstützen die gefundenen Resultate die Gabe von niedrig dosiertem Colchicin bei Patienten mit nichtkardioembolischem ischämischem Schlaganfall oder TIA und sollten in künftigen groß angelegten randomisiert kontrollierten Studien weiter untersucht werden [200]. Die Studiendaten zu Colchicin zur Schlaganfallbehandlung sind in Tab. 7 dargestellt.

**Tab. 7:** *Studiendaten Schlaganfall. *Auflistung von Studien, die den Einsatz von Colchicin zur Prophylaxe und Behandlung von Schlaganfällen untersucht haben unter Berücksichtigung der klinischen Situation, eingesetzten Dosierung, Anwendungsdauer, des Outcomes sowie der häufigsten Nebenwirkung

Studien	Klinische Situation	Dosierung	Anwendungsdauer	Ergebnis	Häufigste UAW unter Colchicin
Nidorf et al. [137]	Stabile KHK	0,5 mg Colchicin/Tag plus Statine und Standardmedikation	36 Monate	Reduktion der Schlaganfallinzidenz unter Colchicin HR 0,22 (95 %-CI 0,02–1,97)	Gastrointestinale Intoleranz bei 2,5 %
Nidorf et al. [134]	Stabile KHK	0,5 mg Colchicin/Tag plus Statine und Standardmedikation	28,6 Monate	Reduktion der Schlaganfallinzidenz unter Colchicin HR 0,66 (95 %-CI 0,35–1,25)	Myalgie bei 21,2 % vs. 18,5 % ohne Colchicin
Tardif et al. [140]	ACS	0,5 mg Colchicin/Tag plus Standardtherapie	20 Monate	Reduktion der Schlaganfallinzidenz unter Colchicin HR 0,26 (95 %-CI 0,10–0,71)	Durchfall bei 17,5 % vs. 17,6 % mit Placebo
Tong et al. [143]	ACS	0,5 mg Colchicin 2‑mal/Tag im ersten Monat gefolgt von 0,5 mg Colchicin/Tag	12 Monate	Reduktion der Schlaganfallinzidenz unter Colchicin HR 0,33 (95 %-CI 0,07–1,66)	Gastrointestinale Intoleranz 23,0 % vs. 20,8 % mit Placebo
Yilmaz et al. [193]	CV-Risiko-Patienten ± Gicht	0,5 mg Colchicin 2‑mal/Tag	6 Monate	Geringere Intima-Media-Dicke unter Colchicin vs. ohne Colchicin (0,98 mm vs. 1,18 mm) Geringes CRP unter Colchicin vs. ohne Colchicin (0,26 mg/dl vs. 0,58 mg/dl)	Nicht erhoben
Yuan et al. [194]	Schlaganfall oder TIA	Gruppe 1: 0,5 mg Colchicin/Tag Gruppe 2: 0,5 mg Colchicin 2‑mal/Tag bis Tag 7 gefolgt von 0,5 mg Colchicin/Tag bis Tag 14 Gruppe 3: 2,0 mg Colchicin-Ladedosis an Tag 1 (aufgeteilt auf 4 Einzeldosen) gefolgt von 0,5 mg Colchicin 2‑mal/Tag bis Tag 7 und 0,5 mg Colchicin/Tag bis Tag 14 Gruppe 4: 3,0 mg Colchicin-Ladedosis an Tag 1 (aufgeteilt auf 6 Einzeldosen) gefolgt von 0,5 mg Colchicin 2‑mal/Tag bis Tag 7 und 0,5 mg Colchicin/Tag bis Tag 14	2 Wochen	Verringertes hsCRP in Gruppe 2 nach 14 Tagen Verringertes hsCRP in den Gruppen 1 und 2 nach 7 Tagen Verringertes hsCRP in den Gruppen 1, 2 und 3 nach 72 h	3 Patienten mit Durchfall (1 aus Gruppe 2 und 2 aus Gruppe 4) 2 Patienten mit Übelkeit bzw. Erbrechen (jeweils 1 aus Gruppe 3 und 4)
Kelly et al. [200]	Schlaganfall oder TIA	0,5 mg Colchicin/Tag plus Standardtherapie	6 Monate	Auftreten des primären Endpunktes (erster tödlicher oder nichttödlicher ischämischer Schlaganfall, MI, Herzstillstand oder Hospitalisierung) in 9,8 % mit Colchicin und 11,7 % ohne Colchicin	Durchfall bei 12,1 % vs. 2,0 % ohne Colchicin

*ACS* akutes Koronarsyndrom, *HR* Hazard Ratio, *hsCRP* hochsensitives C‑reaktives Protein, *MI* Myokardinfarkt, *TIA* transitorisch-ischämische Attacke, *UAW* unerwünschte Arzneimittelwirkung

Folgende große Studien, die sich explizit mit der Wirkung von Colchicin auf das Schlaganfallüberleben beschäftigen, laufen gerade. Ziel der australischen CASPER-Studie (Colchicine After Stroke to Prevent Event Recurrence; ACTRN12621001408875) ist es, die Wirksamkeit von niedrig dosiertem Colchicin (0,5 mg/Tag) zusätzlich zu einer optimalen medizinischen Therapie auf kardiovaskuläre Ergebnisse bei Schlaganfallpatienten mit Anzeichen einer anhaltenden Koronarentzündung (basierend auf hsCRP) zu bewerten. CHANCE‑3 (NCT05439356) schließt über 8000 Hochrisikopatienten mit akutem leichtem bis mittelschwerem ischämischem Schlaganfall oder vorübergehender ischämischer Attacke und einem erhöhten hsCRP ein [201]. Untersucht wird der Effekt von niedrig dosiertem Colchicin (innerhalb der ersten 24 h für insgesamt 90 Tage) als Add-on zur Standardbehandlung auf schwerwiegende unerwünschte kardiovaskuläre Ereignisse gegenüber Placebo [201]. Das Studienende ist für 2025 vorgesehen. RIISC-THETIS vergleicht die Wirksamkeit von Colchicin vs. Placebo sowie Colchicin plus Ticagrelor vs. ASS (2 × 2 faktorielles Design) bei Patienten mit einem ischämischen Schlaganfall mit ipsilateraler atherosklerotischer Stenose bezüglich vaskulärer Ereignisse (NCT05476991). Diese und weitere kommende Studien mit Colchicin sind in Tab. 8 abgebildet. Es bleibt also spannend für den Einsatz von Colchicin zur Schlaganfallprophylaxe.

**Tab. 8:** *Kommende Studien. *Überblick derzeit laufender Studien mit Colchicin einschließlich des primären Endpunktes, der Intervention sowie des voraussichtlichen Studienendes

Indikation	Primärer Endpunkt	Medikation	Voraussichtliches Studienende	Abkürzung	Identifikationsnummer
ACS	MACE	Gruppe 1: 0,5 mg/Tag Colchicin +25 mg/Tag Spironolacton ± SYNERGY-Stent (SYNERGY™ Stentsystem, ©2025 Boston Scientific Corporation, Marlborough, MA, USA) Gruppe 2: 25 mg/Tag Spironolacton ± SYNERGY-Stent Gruppe 3: 0,5 mg/Tag Colchicin ± SYNERGY-Stent Gruppe 4: Placebo ± SYNERGY-Stent	2024-07	CLEAR SYNERGY	NCT03048825
Schlaganfallprophylaxe	Schlaganfall (ischämisch oder hämorrhagisch) innerhalb des Follow-up-Zeitraums von 90 Tagen	1 mg Colchicin in den ersten 3 Tagen, gefolgt von 0,5 mg/Tag Colchicin bis Tag 90 oder Placebo	2024-04	CHANCE‑3	NCT05439356
CV-Primärprophylaxe	Erstes Event, bestehend aus CV-Tod, wiederbelebter Herzstillstand, nichttödlicher MI, nichttödlicher Schlaganfall oder Hospitalisierung aufgrund von Angina pectoris, die eine PCI erfordert	0,5 mg/Tag Colchicin oder 40 mg 2‑mal/Tag Aspirin für 60 Monate oder bis zum ersten Event	2027-12	COLCOT-T2D	NCT05633810
CV-Sekundärprophylaxe	Veränderung in der Aufnahme von FDG ins Gewebe, gemessen als TBR („tissue-to-blood ratio“) im MDS (Maximum Disease Segment) nach 6 Monaten	0,6 mg/Tag Colchicin oder Placebo	2025-08	CADENCE	NCT04181996
Schlaganfall	MACE	0,5 mg/Tag Colchicin oder Placebo für 3 Jahre	2027-12	CASPER	ACTRN12621001408875
Schlaganfall	Anzahl der Patienten mit nichttödlichem Schlaganfall nach 36 bis 60 Monaten	0,6 mg/Tag Colchicin oder Placebo ergänzend zu Best Medical Care	2027-09	RIISC-THETIS	NCT05476991

*FDG* Fluordesoxyglucose, *MACE* „major adverse cardiac events“, *PCI* perkutane Koronarintervention

## Restenosen nach PCI

Perkutane Koronarinterventionen und chirurgische Revaskularisierungen zählen zum therapeutischen Standardrepertoire nach ACS, unabhängig davon, ob es sich um einen STEMI oder NSTEMI handelt [202]. Wiedereröffnete Gefäße werden mit einem Stent versorgt, und eine medikamentöse Prophylaxe, bestehend aus ASS und P2Y_12_-Inhibitor, wird eingeleitet (Klasse-I-Empfehlung) [202]. Dennoch passiert es nach wie vor, dass sich die Gefäße wieder verschließen [203]. Die Mechanismen von (In-Stent‑)Restenosen sind multifaktoriell und für gewöhnlich auf eine übermäßige Gewebeproliferation zurückzuführen [204]. Mit dem Einsatz von medikamentenbeschichteten Stents (DES) bzw. Ballons (DEB) gelingt es, die unerwünschte Zellvermehrung zu hemmen und die Rate von In-Stent-Restenosen verglichen mit den davor gängigen Bare-Metal-Stents (BMS) erheblich zu reduzieren [205]. Betrug die Inzidenz für Restenosen in der Zeit vor Stents noch bis zu 55 %, sank sie nach Einführung der BMS auf rund 17 % und wurde durch DES/DEB auf unter 10 % verringert [206–209].

Auf der Suche nach dem eigentlichen Trigger der Gewebeproliferation geriet die PCI-induzierte endovaskuläre Verletzung ins Visier [210]. Die Vermutung lag nahe, dass die damit einhergehende lokale Entzündungsreaktion als Auslöser der Zellvermehrung fungiert [210]. Diese Entzündungsreaktion äußert sich durch erhöhte hsCRP-Spiegel, die bereits 1 h nach Stentimplantation nachweisbar sind [211]. Mittlerweile ist eindeutig belegt, dass periprozedurale Entzündungen mit schwerwiegenden kardiovaskulären Ereignissen assoziiert sind [212]. Colchicin greift in die Expression und Freisetzung proinflammatorischer Botenstoffe ein und hat das Potenzial, die Proliferation glatter Gefäßmuskelzellen zu stoppen [161]. Zwei ältere Studien, die den Effekt von 2‑mal täglich 0,6 mg Colchicin für 6 Monate auf die Restenoserate nach klassischer Ballonangioplastie ohne Stentimplantation evaluierten, kamen zu einem negativen Ergebnis [213, 214]. In einer jüngeren prospektiven Studie an 196 Patienten mit Diabetes, die sich einer PCI mit BMS unterzogen, konnte 2‑mal täglich 0,5 mg Colchicin für 6 Monate jedoch mit einer geringeren Rate an In-Stent-Restenosen überzeugen (16 % vs. 33 %) [215]. Bei 59 Patienten mit CABG unter Einsatz einer Herz-Lungen-Maschine verminderte 2‑mal täglich 0,5 mg Colchicin über 10 Tage perioperative Myokardschäden, beurteilt anhand der Spitzenkonzentration von hs-Toponin T sowie CK-MB innerhalb von 48 h [216].

Basierend auf diesen Resultaten, wurden die doppelblind-randomisierten Studien COLCHICINE-PCI und COPE-PCI (Colchicine to Prevent Periprocedural Myocardial Injury in PCI) ins Leben gerufen [142, 217]. COLCHICINE-PCI umfasste 400 Patienten mit ACS oder stabiler KHK, die 1–2 h vor einer PCI entweder 1,2 mg Colchicin oder Placebo und unmittelbar nach der PCI 0,6 mg Colchicin oder Placebo erhielten [142]. Zwischen den beiden Gruppen gab es keine signifikanten Unterschiede hinsichtlich PCI-bedingter myokardialer Verletzung (57,3 % vs. 64,2 %), Todes, nichttödlichen Myokardinfarkts und Revaskularisierung des Zielgefäßes nach 30 Tagen (11,7 % vs. 12,9 %) und Outcome eines PCI-bedingten Myokardinfarktes (2,9 % vs. 4,7 %) [142]. Die Colchicin-Therapie korrelierte allerdings mit niedrigeren IL-6- und CRP-Anstiegen [142]. Auch im Follow-up nach 3,3 Jahren unterschied sich die Inzidenz schwerwiegender unerwünschter kardiovaskulärer Ereignisse nicht (32,5 % unter Colchicin vs. 34,9 % unter Placebo) [218]. Dagegen verringerte Colchicin (Initialdosis 1 mg, gefolgt von 0,5 mg 1 h später, 6–24 h vor PCI) in COPE-PCI die Rate periprozeduraler Myokardinfarkte bei Patienten mit NSTEMI oder stabiler Angina pectoris [217]. In der laufenden ORCA-Studie (NCT04382443) wird zurzeit 1 mg Colchicin über 3 Monate plus BMS einer alleinigen DES-Implantation gegenübergestellt [219]. Primärer Outcome-Parameter ist die MACE-Inzidenz im ersten Jahr. Bei positivem Ergebnis wäre die Implantation eines BMS plus Colchicin eine sichere Alternative für Patienten, die für eine DES-Implantation nicht infrage kommen.

## Morbus Behçet

Als Behçet-Syndrom (BS) bezeichnet man eine systemische, autoimmune Entzündung der Blutgefäße [220, 221]. Die Erkrankung ist v. a. im Raum entlang der ehemaligen Seidenstraße verbreitet und manifestiert sich bevorzugt an kleinen Venen und Kapillaren, kann aber grundsätzlich Blutgefäße jeder Größe befallen [220, 221]. Betroffen sind oft die Gefäße der Haut (Papulopusteln, Follikulitis, Erythema nodosum), Schleimhäute (rezidivierende Aphten im Mund- und Genitalbereich), Gelenke (Oligoarthritis, Arthralgien, Sakroiliitis), Augen (Uveitis, Iritis, Hypopyon mit Erblindungsgefahr), das Nervensystem (Neuro-Behçet; Meningoenzephalitis) sowie der Magen-Darm-Trakt (Bauchschmerzen, Durchfall, Ulzera) [222]. Die mannigfaltigen Ausprägungen machen BS zu einer Systemerkrankung mit klinisch sehr variablem Bild [222]. BS hat in der Regel einen schubförmig-remittierenden Verlauf [222]. Ziel der Behandlung ist es, die entzündlichen Exazerbationen zu verhindern und Rezidiven vorzubeugen, um irreversible Organschäden zu verhindern [222].

In der aktiven Phase der Erkrankung sind die Serumspiegel unzähliger proinflammatorischer Botenstoffe, darunter IL‑1, IL‑6, IL‑8, IL-12 und TNF‑α, erhöht [223]. BS zeigt deshalb gutes Ansprechen auf pharmakologische Interventionen mit einer breiten entzündungshemmenden Wirkung wie Colchicin [222]. Das Alkaloid hemmt die Rekrutierung, Adhäsion und Aktivierung von Neutrophilen und unterbricht die Aktivierung des NLRP3-Inflammasoms [223]. Mehrere randomisiert kontrollierte Studien bestätigen die Wirksamkeit von 1–2 mg Colchicin pro Tag auf Geschwüre im Genitalbereich, knotige Läsionen und Gelenkmanifestationen [224–226]. Der Effekt auf papulopustulöse, akneähnliche Läsionen scheint begrenzt zu sein [224–226]. Angesichts der Sicherheit und guten Verträglichkeit von Colchicin wird in den aktuellen EULAR-Empfehlungen auch bei Patienten mit isolierter mukokutaner Beteiligung, die auf topische Maßnahmen wie Steroide nicht ansprechen, ein Therapieversuch mit Colchicin befürwortet, insbesondere wenn es sich bei der dominanten Läsion um ein Erythema nodosum oder ein Genitalgeschwür handelt (Evidenzgrad IB; Empfehlungsstärke: A) [227]. Des Weiteren ist Colchicin das Mittel der ersten Wahl bei BS-Patienten mit akuter Arthritis (Evidenzgrad IB; Empfehlungsstärke: A) [227].

## Chronisch kutane Vaskulitis

**Abb. 4:**
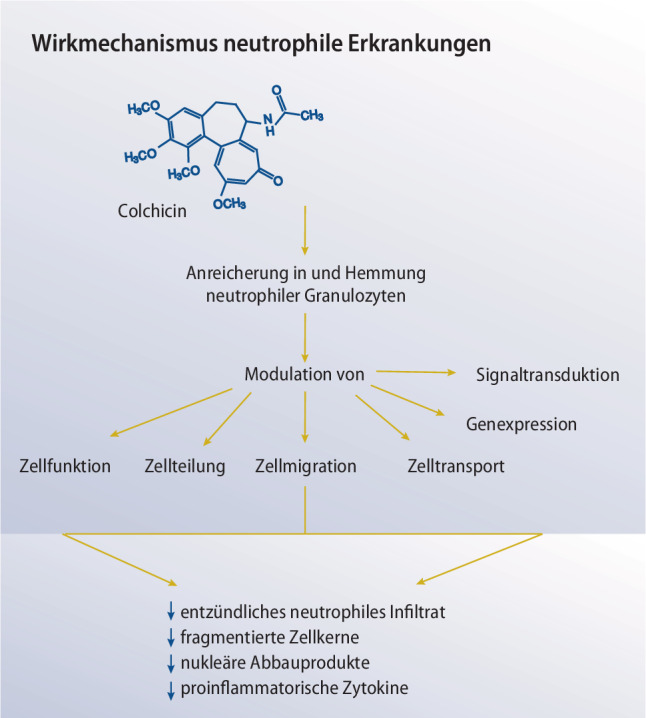
*Wirkungsmechanismus bei neutrophilen Erkrankungen. *Colchicin wird im Körper reichlich von Leukozyten aufgenommen. Auf diese Weise moduliert es nachhaltig zahlreiche zelluläre Funktionen wie die Genexpression, Signaltransduktion, Zellteilung und Migration. Colchicin greift damit ganz am Beginn der pathophysiologischen Signalkaskade neutrophiler Erkrankungen ein

Die kutane Vaskulitis ist eine Gefäßerkrankung, die an den kleinen und mittelgroßen Gefäßen (Arteriolen, Kapillaren, postkapilläre Venolen) der Haut und des subkutanen Gewebes in Erscheinung tritt, ohne Befall der inneren Organe [228]. Die Diagnose wird mittels Biopsie gestellt [229]. Richtungsweisende Symptome einer kutanen Vaskulitis beinhalten Purpura, Petechien und flache Ulzera [229]. Eine Sonderform, die sich histopathologisch durch ein entzündliches Infiltrat aus Neutrophilen mit fibrinoider Nekrose, Fragmentierung der Zellkerne und Ablagerung nukleärer Abbauprodukte in der Gefäßwand präsentiert, ist die leukozytoklastische Vaskulitis [230]. Ihre Therapie hängt von der Ätiologie und dem Ausmaß der Erkrankung ab [230]. Lässt sich keine Ursache finden, ist Colchicin, das nachhaltig die Funktion neutrophiler Granulozyten moduliert und insofern ganz am Beginn der pathophysiologischen Signalkaskade eingreift (vgl. Abb. 4), eine effektive Behandlungsoption [38, 223, 231, 232]. In Fallberichten und einer kleinen randomisiert kontrollierten Studie verbesserte Colchicin in einer Dosierung von 0,5 bzw. 0,6 mg 2‑mal täglich die Haut- und Gelenksymptome und führte bei manchen Patienten zu einer vollständigen Abheilung der Hautläsionen [231, 232]. Colchicin ist demnach eine wirksame und sichere Alternative bei Patienten mit kutaner Vaskulitis, die auf systemische Kortikosteroide nicht ansprechen, diese nicht vertragen, bei denen Kontraindikationen bestehen oder die Kortikosteroide generell ablehnen.

## Sweet-Syndrom

Das – wie Morbus Behçet, Pyoderma gangraenosum und die neutrophile ekkrine Hidradenitis zu den neutrophilen Dermatosen gerechnete – Sweet-Syndrom weist eine zu diesen Entitäten überlappende Pathophysiologie, bestehend aus einer autoinflammatorischen Komponente mit überwiegend neutrophilem Infiltrat, auf [233]. Klinisch ist das Sweet-Syndrom durch Fieber, Neutrophilie und erythematöse Hautläsionen gekennzeichnet [234]. Papeln, Knötchen und Plaques sind oft asymmetrisch verteilt und gemeinhin im Gesicht, am Hals und den oberen Extremitäten lokalisiert [235]. Die betroffenen Stellen zeigen histologisch ein charakteristisches diffuses neutrophiles Infiltrat in der oberen Dermis [235]. Das Sweet-Syndrom tritt in 3 klinischen Situationen auf: klassisch (idiopathisch), malignitätsbedingt und medikamenteninduziert [236]. Die klassische Variante betrifft fast immer Frauen im Alter zwischen 30 und 50 Jahren und hat eine hohe Rezidivrate [236]. Lymphozyten, im Speziellen T‑Helfer-Zellen vom Typ 1 (Th1), scheint eine wichtige Rolle beim Auftreten des klassischen Phänotyps innezuwohnen, da diese Zellpopulation für die Aktivierung und Lokalisierung der Neutrophilen verantwortlich ist [237]. Das wird durch erhöhte Serumspiegel von Th1-Zytokinen (IL-1α, IL-1β, IL‑2 und IFN-γ) an Patienten mit Sweet-Syndrom belegt und durch das klinische Ansprechen auf Colchicin untermauert, das an mehreren Stellen in die Zellfunktion, -teilung und -migration, Signaltransduktion, Genexpression und den Zelltransport von Leukozyten eingreift (vgl. Abb. 4; [237, 238]).

Suehisa und Tagami waren 1981 die ersten, die über die Wirksamkeit von Colchicin beim Sweet-Syndrom bei einem 29-jährigen Mann berichteten [239]. Im Jahr 1983 folgte ein weiterer Fallbericht im *British Journal of Dermatology* über 3 weitere Patienten [240]. Seitdem wurden mehrere Fallberichte und größere, zumeist retrospektive Studien veröffentlicht, die ein rasches Ansprechen auf 2‑ bis 3‑mal täglich 0,5 mg Colchicin sowohl in der Akut- als auch in der Erhaltungstherapie des Sweet-Syndroms demonstrieren [241–249]. Fieber, Hautläsionen, Arthralgien und Leukozytose normalisierten sich nach kurzer Zeit bei einer mittleren Behandlungsdauer von 2 Wochen [241–249].

## Diabetes mellitus

Diabetes mellitus, im Besonderen Typ-2-Diabetes (T2D), ist ein Hauptrisikofaktor für Herz-Kreislauf-Erkrankungen [250]. Für Erwachsene mit Diabetes dokumentiert die Framingham-Studie eine Prävalenz von 75–85 % für Bluthochdruck, 70–80 % für erhöhtes LDL‑C und 60–70 % für Fettleibigkeit [251]. Das „tödliche Quartett“ aus Insulinresistenz, atherogener Dyslipidämie, bauchbetonter Adipositas und Bluthochdruck hat unter dem Namen metabolisches Syndrom Eingang in die wissenschaftliche Literatur gefunden [252]. Ein gemeinsamer Nenner der 4 Krankheitsbilder ist ein chronisches, geringgradiges Entzündungsgeschehen („silent inflammation“) [253].

Wissenschaftler sind überzeugt, dass lokale Entzündungen in der Gefäßwand eine entscheidende Funktion beim Fortschreiten der Atherosklerose und dem Aufbrechen instabiler Plaques einnehmen [254]. Beschädigte vaskuläre Endothelzellen werden aktiviert und steigern die Bildung und Freisetzung von Adhäsionsmolekülen, die ihrerseits zirkulierende Leukozyten anlocken, die sich der Oberfläche anhaften und in die innerste Gefäßschicht wandern [107]. Migrierte Leukozyten differenzieren sodann zu Makrophagen, nehmen oxidiertes LDL über den Scavenger-Rezeptor auf und sezernieren entzündliche Zytokine [107]. Hinweise, wonach die beschriebenen Mechanismen Atherosklerose, Plaquedestabilisierung und Thrombosen begünstigen, machen Leukozyten zu einem potenziellen therapeutischen Ziel, um Herz-Kreislauf-Erkrankungen vorzubeugen [107]. Colchicin reichert sich in Leukozyten an und unterdrückt deren Aktivierung, indem es den Aufbau von Mikrotubuli im Zytoskelett inhibiert [102]. Die Hemmung umfasst auch das NLRP3-Inflammasom, was Colchicin zu einem aussichtsreichen Kandidaten zur Behandlung von mit vaskulären Entzündungsreaktionen vermittelten Krankheitsbildern macht [42, 255].

In LoDoCo2 senkte niedrig dosiertes Colchicin (0,5 mg/Tag) das kardiovaskuläre Risiko bei Patienten mit chronischer KHK, was in der Zulassung von Colchicin für diese Indikation durch die FDA mündete [122, 134]. Eine jüngere Analyse konzentrierte sich auf die Wirksamkeit von Colchicin bei Patienten mit KHK und Diabetes (18,2 % der LoDoCo2-Studienpopulation) und versuchte herauszufinden, ob Colchicin die Entwicklung eines neu auftretenden T2D verzögert [256]. Für den primären Endpunkt kardiovaskulärer Tod, spontaner Myokardinfarkt, ischämischer Schlaganfall oder durch Ischämie verursachte Revaskularisation gab es keine Unterschiede zwischen Patienten mit oder ohne Diabetes [256]. Die Inzidenz für einen neu auftretenden T2D lag bei 1,5 % im Colchicin- und 2,2 % im Placeboarm – ein vorbeugender Effekt, der in größeren prospektiven Studien bestätigt werden muss [256]. Rezente aus der COLCOT-Studie entnommene Daten von 959 Patienten mit T2D, die niedrig dosiertes Colchicin innerhalb von 30 Tagen nach einem ACS erhalten hatten, zeigen, dass Colchicin die Rate an kardiovaskulären Ereignissen gegenüber Placebo signifikant reduziert (8,7 % vs. 13,1 %) [10].

Die Primärprävention mit Colchicin wird gerade in COLCOT-T2D untersucht (NCT05633810). Eine japanische Phase-2-Studie setzt einen Schritt weiter vorher an und eruiert die optimale Colchicin-Dosis bei Patienten mit KHK und T2D in Bezug auf den inflammatorischen Marker hsCRP [257]. Die Ergebnisse der Studie werden für das Protokoll einer künftigen Phase-3-Studie herangezogen, deren primärer Endpunkt die KHK-Reduktion ist [257]. Erste Ergebnisse der Canadian Study of Arterial Inflammation in Patients With Diabetes and Vascular Events: EvaluatioN of Colchicine (CADENCE; NCT04181996) werden für 2025 erwartet [258]. Hauptziel von CADENCE besteht darin, die Wirkung von Colchicin bei Patienten mit T2D oder Prädiabetes, mit einem kürzlich zurückliegenden kardiovaskulären Ereignis (ACS, Schlaganfall oder TIA), auf Gefäßentzündungen der Halsschlagadern und aufsteigenden Aorta zu bewerten [258]. Die Effektivität der Colchicin-Therapie wird mittels PET-CT quantifiziert [258].

## Zusammenfassung und Ausblick

Colchicin ist ein natürlicher pflanzlicher Inhaltsstoff der Herbstzeitlosen und eines der ältesten Arzneimittel, das immer noch verwendet und umfassend erforscht wird. Seinen einzigartigen Wirkungsmechanismus macht man sich bei unzähligen Krankheitsbildern zunutze, die eine entzündlich-neutrophile Komponente aufweisen (vgl. Tab. 9). Randomisiert kontrollierte Studien konnten zuletzt eindrücklich die Wirkung und gute Verträglichkeit von niedrig dosiertem Colchicin bei kardiovaskulären Erkrankungen darlegen, und das unabhängig von einer bestehenden Statin- und thrombozytenaggregationshemmenden Therapie. Colchicin füllt damit eine therapeutische Lücke, ohne den Blutdruck, das Blutungsrisiko oder den Herzrhythmus zu beeinflussen. In den nächsten Jahren werden COLCOT-T2D, CLEAR SYNERGY, CASPER, CADENCE und CHANCE-3 weitere Erkenntnisse zur Wirksamkeit und Verträglichkeit von Colchicin bei kardiovaskulären Risikopatienten liefern.

**Tab. 9:** *Evidenzbasierter Einsatz von Colchicin. *Etablierte und mögliche zukünftige Indikationsgebiete von Colchicin

Hohe Evidenz für Nutzen. In Leitlinien empfohlen	Moderate/geringe Evidenz für Nutzen. Keine Empfehlung in Leitlinien	Hinweise auf Nutzen. Könnte künftig eine Rolle spielen
Gicht	Akutes Koronarsyndrom	Herzinsuffizienz
Familiäres Mittelmeerfieber	Vorhofflimmern	Restenosen nach PCI
Akute/rezidivierende Perikarditis	Schlaganfall	Typ-2-Diabetes
Dressler-Syndrom	Chronisch kutane Vaskulitis	–
Chronische KHK	Sweet-Syndrom	–
Morbus Behçet	Neutrophile Erkrankungen	–
